# Multiple Drivers of High Species Diversity and Endemism Among *Alyssum* Annuals in the Mediterranean: The Evolutionary Significance of the Aegean Hotspot

**DOI:** 10.3389/fpls.2021.627909

**Published:** 2021-04-27

**Authors:** Veronika Cetlová, Judita Zozomová-Lihová, Andrea Melichárková, Lenka Mártonfiová, Stanislav Španiel

**Affiliations:** ^1^Institute of Botany, Plant Science and Biodiversity Centre, Slovak Academy of Sciences, Bratislava, Slovakia; ^2^Botanical Garden of P. J. Šafárik University in Košice, Košice, Slovakia; ^3^Department of Botany, Faculty of Science, Charles University, Prague, Czechia

**Keywords:** Aegean area, allopolyploidy, *Alyssum*, annual species, endemics, Mediterranean, phylogeny, sympatry

## Abstract

The Mediterranean Basin is a significant hotspot of species diversity and endemism, with various distribution patterns and speciation mechanisms observed in its flora. High species diversity in the Mediterranean is also manifested in the monophyletic lineage of *Alyssum* annuals (Brassicaceae), but little is known about its origin. These species include both diploids and polyploids that grow mainly in open and disturbed sites across a wide elevational span and show contrasting distribution patterns, ranging from broadly distributed Eurasian species to narrow island endemics. Here, we investigated the evolution of European representatives of this lineage, and aimed to reconstruct their phylogeny, polyploid and genome size evolution using flow cytometric analyses, chloroplast and nuclear high- and low-copy DNA markers. The origin and early diversification of the studied *Alyssum* lineage could be dated back to the Late Miocene/Pliocene and were likely promoted by the onset of the Mediterranean climate, whereas most of the extant species originated during the Pleistocene. The Aegean region represents a significant diversity center, as it hosts 12 out of 16 recognized European species and comprises several (sub)endemics placed in distinct phylogenetic clades. Because several species, including the closest relatives, occur here sympatrically without apparent niche differences, we can reject simple allopatric speciation via vicariance as well as ecological speciation for most cases. Instead, we suggest scenarios of more complex speciation processes that involved repeated range shifts in response to sea-level changes and recurrent land connections and disconnections since the Pliocene. In addition, multiple polyploidization events significantly contributed to species diversity across the entire distribution range. All seven polyploids, representing both widespread species and endemics to the western or eastern Mediterranean, were inferred to be allopolyploids. Finally, the current distribution patterns have likely been affected also by the human factor (farming and grazing). This study illustrates the complexity of evolutionary and speciation processes in the Mediterranean flora.

## Introduction

The Mediterranean Basin represents one of the world’s major biodiversity hotspots with an exceptionally high species diversity and endemism rate (Myers et al., 2000; Thompson, 2020). This species richness can be attributed to a variety of different factors. Complex geological history, high topographical and ecological heterogeneity of this region have promoted allopatric and ecological diversification and speciation (Médail and Diadema, 2009; Hewitt, 2011; Nieto Feliner, 2014; Thompson, 2020). In addition, the impact of Quaternary climatic oscillations was less severe in the Mediterranean than in central and northern Europe, allowing preservation and accumulation of diversity through small-scale range shifts with minimized population and species extinctions (Nieto Feliner, 2011). Several major paleoclimatic and paleogeologic events have exerted crucial influences on Mediterranean flora, especially the Mediterranean Sea desiccation during the Messinian salinity crisis (ca. 5.96–5.33 mya; Krijgsman et al., 1999), the onset of the Mediterranean climate (ca. 3.2 mya, Suc, 1984), and sea-level oscillations that occurred during the glacial and interglacial periods of the Pleistocene (Lambeck et al., 2014). Sea-level drops resulted in the formation of temporary land connections between major peninsulas (e.g., between the Balkan and Apennine Peninsulas, Correggiari et al., 1996), between islands, and between the mainland and islands (e.g., Anatolia and East Aegean islands, Simaiakis et al., 2017). At the latest Pleistocene, the Aegean and Ionian sea level was ca. 120 m lower than today, and Aegean islands formed a kind of land bridges connecting mainland Greece and Turkey, separated locally by sea channels (Perissoratis and Conispoliatis, 2003), which enhanced plant migrations. Sea-level oscillations also fostered distribution range shifts, which led to secondary contacts and gene flow between otherwise isolated species and lineages (Nieto Feliner, 2011, 2014).

The Mediterranean Basin and the adjacent mountain ranges represent the center of species diversity for *Alyssum* L. (Dudley, 1965; Ball and Dudley, 1993; Jalas et al., 1996), which is the largest genus in the tribe Alysseae and comprises approximately 114 species, including 30 annuals and 84 perennials (Španiel et al., 2015, but still subject of taxonomic revisions). The genus *Alyssum*, in its traditional taxonomic treatment, was nearly twice as large (Al-Shehbaz, 2012); however, many species have recently been transferred into the genera *Cuprella* Salmerón-Sánchez, Mota & Fuertes, *Meniocus* Desv., and *Odontarrhena* C.A.Mey. ex Ledeb. (Španiel et al., 2015). In the current delimitation, the genus *Alyssum* includes three of its six original sections (Dudley, 1964b): *Alyssum* sect. *Alyssum*, *A.* sect. *Gamosepalum* (Hausskn.) T.R.Dudley, and *A.* sect. *Psilonema* (C.A.Mey.) Hook.f. Recent phylogenetic analyses, however, have revealed two major clades in *Alyssum* s.str., which do not agree with the traditional sectional classification (Rešetnik et al., 2013; Li et al., 2015; Salmerón-Sánchez et al., 2018). One clade includes annual species of *A.* sect. *Psilonema* and both annual and perennial species of *A.* sect. *Alyssum*, whereas the other clade includes perennial species from *A.* sect. *Alyssum* and *A.* sect. *Gamosepalum* and an annual species *Alyssum dasycarpum* Stephan ex. Willd. from *A.* sect. *Psilonema.* The present study focuses on the former clade, which comprises all annuals from sections *Alyssum* and *Psilonema* (except for the phylogenetically distant *A. dasycarpum*) and a nested *Alyssum montanum–Alyssum repens* perennial species complex. Whereas the taxonomy and evolutionary history of the *A. montanum–A. repens* species complex have been thoroughly explored in a series of recent studies (e.g., Španiel et al., 2011, 2017a,b, 2019; Magauer et al., 2014; Zozomová-Lihová et al., 2014, 2020; Arrigo et al., 2016; Melichárková et al., 2017), little is known about the phylogenetic relationships and speciation processes of the annual taxa. Here, we attempted to fill this gap by exploring the evolutionary history of all 16 annual taxa of *Alyssum* reported in Europe, some of which extend also to northern Africa or Asia (see Table 1). Altogether, the studied clade comprises tentatively 29 annual species, thus about 13 extra-European annuals distributed mainly in the Irano-Anatolian area, Caucasus and Central Asia (Dudley, 1964a,b, 1965; Rechinger, 1968) were not included in our study. Their circumscription and taxonomic treatment is namely uncertain in several cases, and detailed field and herbarium research is needed before including them in phylogenetic studies, which was beyond the scope of the present study.

**TABLE 1:** Overview of the studied *Alyssum* annual species (listed alphabetically), including their distribution ranges, habitat characteristics and site coexistence with other species.

Species	Distribution	Area codes	Habitat	Site coexistence
*A. alyssoides* (L.) L.	Europe; N Africa; SW, C Asia	ABCEFGHIJ	Steppes, rocky slopes, quarries, disturbed ground, pastures, roadsides; limestone, but also other substrates (schist, flysch, serpentine), 0–2,300 m a.s.l.	*hirsutum, minutum, simplex, turkestanicum, umbellatum*
*A. collinum* Brot.	SW Europe; N Africa	FJ	Pastures, steppes, open dry sites, cultivated areas, roadsides, gravel, screes, rocks, sands; calcareous and other substrates, 50–1,850 m a.s.l.	*granatense*
*A. foliosum* Bory & Chaub.	GR-m, Ae; MK; CY; wTR	ABCE	Less exposed microsites between rocks or spiny shrubs in dry rocky scrubland, screes, pastures; limestone, 400–1,800 m a.s.l.	*fulvescens, minutum, simplex, simulans, smyrnaeum, xiphocarpum*
*A. fulvescens* Sm.	GR-Ae; wTR; ?CY	AB	Rocky and gravelly ground in open scrubland, pastures and rocky slopes; limestone (in Turkey reportedly also on volcanic substrates and sand dunes), 20–1,300 m a.s.l.	*foliosum, simplex, smyrnaeum, umbellatum*
*A. granatense* Boiss. & Reut.	SW Europe; N Africa	FJ	Gravelly pastures, open disturbed ground, roadsides; calcareous substrates, schist, granite, 40–2,700 m a.s.l.	*collinum, minutum*
*A. hirsutum* M.Bieb.	E, SE Europe; TR	BEH	Seashores, sandy, rocky or gravelly places, riverbeds, ruderal sites, cultivated areas; mainly calcareous substrates, 0–1,300 m a.s.l.	*alyssoides, minutum, turkestanicum*
*A. minutum* Schltdl. ex DC.	S, E Europe (incl. Ae); N Africa; SW Asia	ABCDEF*G*H*IJ	Steppes, open ground, dry ruderal sites, sandy seashores, gravelly pastures; mainly limestone, 0–2,700 m a.s.l.	*alyssoides, foliosum, granatense, hirsutum, siculum, simplex, simulans, smyrnaeum, umbellatum, xiphocarpum*
*A. pogonocarpum* Carlström	GR-Ae (Rhodos); sTR, ?cTR	AB	Ultramafic (serpentine) gravelly slopes, ca. 250 m a.s.l.	*simplex, strigosum*
*A. siculum* Jord.	GR-m, Ae (Crete); IT (Sicily)	ACD	Rocky slopes, gravel, open disturbed ground, pastures; mainly limestone, 600–2,200 m a.s.l.	*minutum, simplex*
*A. simplex* Rudolphi	S, E Europe (incl. Ae); SW, C Asia; CY; ?N Africa	ABCDEFGHI(?J)	Pastures, open ground, rocky and gravelly slopes; calcareous and other substrates (including serpentine), 0–2,000 m a.s.l.	*alyssoides, foliosum, fulvescens, minutum, pogonocarpum, siculum, simulans, smyrnaeum, strigosum*
*A. simulans* Runemark ex Hartvig	GR-m, Ae (Crete)	AC	Rocky slopes, screes, open disturbed ground, pastures; limestone, 600–1,900 m a.s.l.	*alyssoides, foliosum, minutum, siculum, simplex, smyrnaeum*
*A. smyrnaeum* C.A.Mey.	GR-m, Ae; TR; Crimea	ABC	Rocky slopes, screes, dry grasslands; calcareous substrates, 150–1,400 m a.s.l.	*foliosum, fulvescens, minutum, simplex, simulans*
*A. strigosum* Banks & Sol.	S Europe (incl. Ae); SW Asia; CY; ?N Africa	ABCEFGHI(?J)	Steppes, gravel, fallow fields; calcareous and other substrates (including serpentine), 0–1,200 m a.s.l.	*alyssoides, pogonocarpum, simplex, turkestanicum*
*A. szovitsianum* Fisch. & C.A.Mey.	SW Asia	I	Dry rocky sites, open ground, cultivated areas, 200–2,800 m a.s.l.	
*A. turkestanicum* Regel & Schmalh. (including *A. desertorum* Stapf)	C, E, SE Europe (incl. Ae); SW, C, E Asia;	ABCEHI	Steppes, rocky and sandy places, dry ruderal sites, pastures; mainly limestone and serpentine, 0–2,000 m a.s.l.	*alyssoides, hirsutum, simplex, strigosum*
*A. umbellatum* Desv.	S, E Balkan (incl. Ae); CY; Crimea; wTR; wSY	ACEI	Steppes, dry ruderal sites, gravel roads, riverbeds, seashores; a variety of substrates (including serpentine), 0–900 m a.s.l.	*alyssoides, foliosum, fulvescens, minutum*
*A. xiphocarpum* P. Candargy	GR-Ae (Lesvos)	A	Rocky slopes, dry rocky scrubland; limestone, ca. 730 m a.s.l.	*foliosum, minutum*

*Geographic distributions are reported according to Dudley (1965), Küpfer and Nieto Feliner (1993), Rybinskaya (1994), Jalas et al. (1996), Zhou et al. (2001), Hartvig (2002), Al-Shehbaz (2010), Marhold (2011), Strid (2016), Rešetnik and Španiel (2018), and Cetlová et al. (2019). In the Distribution column, country abbreviations follow standardized two-letter codes (GR, Greece; MK, North Macedonia; CY, Cyprus; TR, Turkey; IT, Italy; SY, Syria); abbreviations ‘w’ and ‘s’ stand for western and southern parts, respectively, and distribution in Greece is given separately for the mainland (‘m’) and Aegean islands (‘Ae’). Species distribution is summarized also in the column Area codes: A*, A*egean islands; B, Turkey (mainland); C, Greece (mainland); D, Sicily; E, Balkan; F, southwestern Europe (Iberian Peninsula, France); G, Apennine Peninsula; H, central and eastern Europe; I, Asia (except Turkey); J, northern Africa. Asterisks given for A. minutum indicate some exceptions: absent from central Europe, France and very rare in the Apennine Peninsula. Habitat data are based on Küpfer and Nieto Feliner (1993) and Hartvig (2002) and our own field observations. Data on site coexistence are based on our field observations, exceptionally on literature sources (site coexistence of A. pogonocarpum with A. strigosum in Rhodos according to Hassler, 2004-2021). For the list and details of the analyzed populations, see Supplementary Table 1.*

The here studied *Alyssum* annuals display contrasting distribution patterns in Europe, ranging from narrow endemics to widespread species (Table 1). Much species diversity is concentrated in the Aegean islands and adjacent mainland areas (Dudley, 1965; Hartvig, 2002; Strid, 2016). All these species grow preferentially on open, disturbed ground and, therefore, can be found on pastures, gravelly screes, rocky or stony slopes, phrygana, macchia, and typically (but not exclusively) on limestone at various elevations. Some species are more ubiquitous (e.g., *Alyssum alyssoides* and *Alyssum minutum*), whereas others are typically present in particular types of habitats (see Table 1 for details). Quite commonly three or even more annual *Alyssum* species can grow in the same locality within a close distance of few meters (field observations and the present sampling, see Table 1 and Supplementary Table 1). Small yellow flowers arranged in racemes are visited by a variety of insects, but nothing is known about the mating system of these *Alyssum* species. Phenological shifts observed between some co-occurring species suggest the existence of premating isolating barriers. Seed dispersal occurs primarily through gravity and zoochory, especially sheep and goats, as the seeds become mucilaginous under wet conditions and easily attach to the animal body (field observations). Three different ploidy levels were previously reported for these annuals, ranging from diploids to hexaploids (Table 2, Španiel et al., 2015).

**TABLE 2:** Chromosome numbers, ploidy level data and relative genome size values of the studied *Alyssum* species.

Species	Chrom. number	Ploidy level	2C ± *SD*	C*x* ± *SD*
*A. alyssoides*	2*n* = 32*^a^	4*x*	0.699 ± 0.011	0.175 ± 0.003
*A. collinum*	2*n* = 32*	4*x*	1.094 ± 0.012	0.273 ± 0.003
*A. foliosum*	2*n* = 16*	2*x*	0.507 ± 0.010	0.253 ± 0.005
*A. fulvescens*	2*n* = 16*^b^	2*x*	0.583 ± 0.020	0.292 ± 0.010
*A. granatense*	2*n* = 48	6*x*	0.966 ± 0.027	0.161 ± 0.004
*A. hirsutum*	2*n* = 46, 48*^c^	6*x*	1.991 ± 0.045	0.332 ± 0.008
*A. minutum*	2*n* = 16*	2*x*	0.496 ± 0.006	0.248 ± 0.003
*A. pogonocarpum*	2*n* = 16	2*x*	0.948 ± 0.011	0.474 ± 0.005
*A. siculum*	2*n* = 48*	6*x*	1.229 ± 0.018	0.205 ± 0.003
*A. simplex*	2*n* = 16*^d^	2*x*	0.602 ± 0.008	0.301 ± 0.005
*A. simulans*	2*n* = 32*	4*x*	1.015 ± 0.032	0.254 ± 0.008
*A. smyrnaeum*	2*n* = 16*	2*x*	0.508 ± 0.010	0.253 ± 0.005
*A. strigosum*	2*n* = 16*^e^	2*x*	0.617 ± 0.007	0.308 ± 0.003
*A. szovitsianum*	2*n* = 16^f^	2*x*	0.505 ± 0.002	0.253 ± 0.001
*A. turkestanicum*	2*n* = 32*^g^	4*x*	0.713 ± 0.009	0.178 ± 0.002
*A. umbellatum*	2*n* = 14*, 16	2*x*	0.701 ± 0.015	0.351 ± 0.007
*A. xiphocarpum*	–	2*x*	0.770 ± 0.010	0.385 ± 0.005

*Data on chromosome numbers include both previously published records (the original literature sources are listed in AlyBase, Španiel et al., 2015) and those determined in the present study (marked by asterisks, see Supplementary Table 1). Superscripts a–g indicate some previously published deviating, rare and/or probably erroneous records (see below). Data on ploidy level and relative genome size values are based on the present flow-cytometric (DAPI) measurements, listed as species-level averages of 2C (total) and Cx (monoploid) values given in arbitrary units ± standard deviation (SD). For the details of the analyzed populations and the population-level genome size values, see Supplementary Table 1. ^*a*^Rare and probably erroneous records of 2n = 16 (Crete) and of 2n = 24 (Italy). ^*b*^A single record of 2n = 32 reported from Turkey, needs verification. ^*c*^A single record of 2n = 16 from Georgia, needs verification. ^*d*^A doubtful record of 2n = 24 from Iran; rare records of 2n = 32 from Iran and Tajikistan. ^*e*^A doubtful record of 2n = 64 from Turkey. ^*f*^Rare and probably erroneous records of 2n = 14, 32 from Iran. ^*g*^Rare and doubtful records of 2n = 16, 24, 48 from Iran and Afghanistan.*

Some hypotheses regarding species relationships can be drawn from morphological resemblance and the previously published phylogeny of Alysseae (Rešetnik et al., 2013). For example, available data suggest that the hexaploid species *Alyssum siculum* is either an allopolyploid derived from the tetraploid *A. alyssoides* and the diploid *Alyssum simplex* or an autopolyploid of *A. alyssoides* (Persson, 1971; Rešetnik et al., 2013). Another hexaploid taxon, *A. granatense*, morphologically resembles the tetraploid *A. alyssoides* (Küpfer and Nieto Feliner, 1993). Morphological similarity indicates close evolutionary relationships between the hexaploid *Alyssum hirsutum* and the diploid *Alyssum pogonocarpum* (Carlström, 1984), between the diploids *A. simplex* and *Alyssum strigosum*, and between the diploids *Alyssum fulvescens* and *Alyssum smyrnaeum* (Hartvig, 2002). The tetraploid *Alyssum simulans* is a suspected allopolyploid of the diploids *Alyssum foliosum* and *A. minutum*, due to its intermediate morphology (Hartvig, 2002). *Alyssum xiphocarpum* (the ploidy level of which has not yet been published) is morphologically very similar to the sympatric diploid *Alyssum umbellatum* and, therefore, has been treated by many authors as a synonym of the latter species (e.g., Dudley, 1965; Jalas et al., 1996); however, *A. xiphocarpum* exhibits much larger petals, anthers and styles than *A. umbellatum* (Hartvig, 2002). In contrast, the tetraploid *Alyssum turkestanicum* does not show obvious morphological affinity to any particular European species, and its phylogenetic relationships are unknown.

In the present study, we aimed to resolve the evolutionary history of all 16 European *Alyssum* annuals described above. To accomplish this goal, we performed the following: (1) assessed the ploidy levels and genome size variation within and among the species; (2) inferred phylogenetic relationships among these species based on multilocus DNA data, including divergence time estimates; (3) proposed scenarios of speciation events based on distribution, phylogenetic, and polyploidy patterns; and (4) formulated consistent hypotheses for the origin of polyploids.

## Materials and Methods

### Plant Material

**FIGURE 1:**
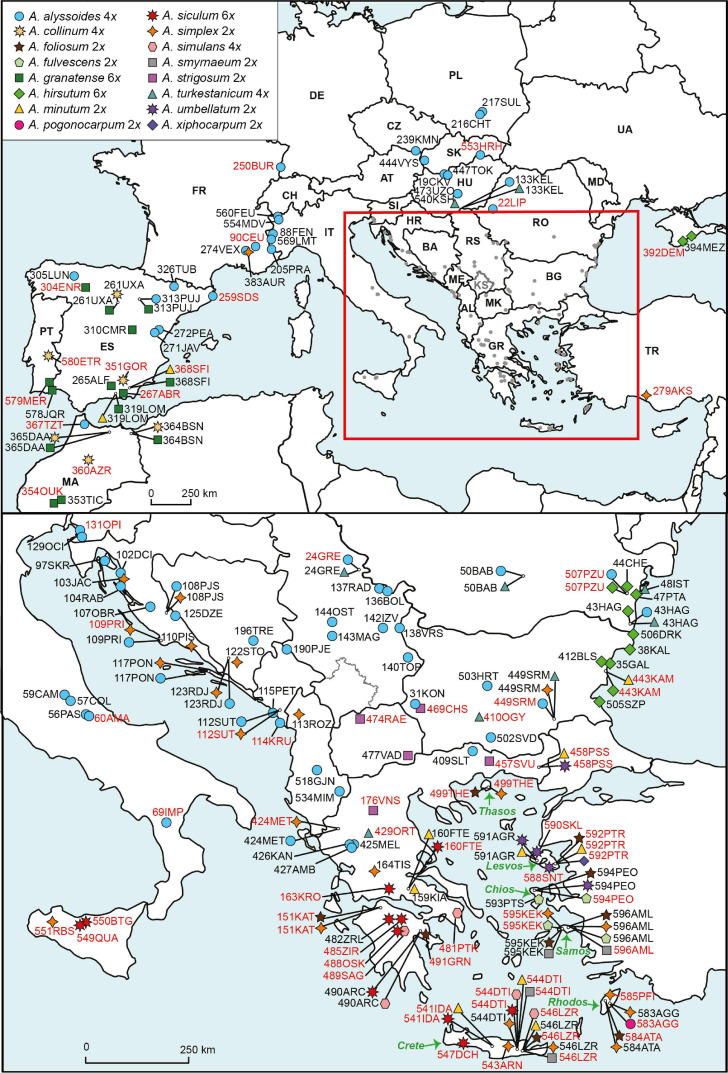
Map of the sample sites of the analyzed annual *Alyssum* species. Population assignments to the species are indicated by different symbol colors and shapes. Ploidy levels are indicated next to the species names. *Alyssum szovitsianum*, which was represented by one population sample (289TRJ-*sz*) from Iran, is not shown on the map. Population codes follow Supplementary Table 1 and are written in either black script (population samples used only for the FCM analyses) or red script (population samples used for both FCM and molecular analyses).

Material sampling comprised all 16 annual *Alyssum* species reported in Europe (Table 1 and Supplementary Table 1). In addition, two species from the well-explored perennial *A. montanum-A. repens* species complex, which is nested as a monophyletic clade within the lineage of the studied annuals, and one southwestern Asian annual species, *Alyssum szovitsianum*, were included. On the other hand, *A. dasycarpum*, which is an annual species that also occurs in Europe but is phylogenetically unrelated to the lineage being explored here (see section “Introduction” and Rešetnik et al., 2013) was not investigated. The sampling focused on the European Mediterranean, and particularly Greece and the Aegean area, which represent the regions with the highest species diversity of *Alyssum* annuals within Europe (Table 1). Two species of the related genus *Odontarrhena* were used as outgroups in the molecular analyses. In total, 191 populations of annual species were sampled and used for flow-cytometric (FCM) analyses. Of these, 76 populations, representing all sampled 17 annual species, were subjected to molecular analyses. A full list of the population samples, including detailed descriptions of their localities, can be found in Supplementary Table 1 and is shown on the map in Figure 1. Tissue samples were collected from five to ten individuals in each population. Leaves and sterile shoots were dried and preserved in silica gel for further use in FCM and DNA extraction procedures. All sampled individuals were analyzed by FCM, and typically, two individuals per population were subjected to molecular analyses. In several cases, when individuals at the sampled localities were in the fruiting stage, or the leaves were withered or damaged, we collected seeds. These seeds were later sown, and the plantlets that sprouted from the seeds were used to obtain vegetative tissues for FCM and molecular analyses. In addition, seeds were collected from most species (whenever available at localities) for chromosome counting. Voucher specimens of the plants analyzed during the present study have been deposited in the herbarium of the Institute of Botany, SAS, Bratislava, Slovakia (SAV).

### Chromosome Counting and Flow Cytometry

Chromosome numbers were determined in the mitotic metaphases of cells from root tips, following the protocol described in Španiel et al. (2018). FCM was used to determine relative genome sizes and to infer the ploidy levels of the investigated annual taxa and populations. Each sampled individual was analyzed separately, measured together with an internal standard (see below). Ploidy levels were inferred by comparing the relative genome size of each sample with the values measured in conspecific plants with known, counted chromosome numbers. In the case of four taxa for which we were unable to obtain chromosome numbers (*A. granatense*, *A. pogonocarpum*, *A. szovitsianum*, and *A. xiphocarpum*), their ploidy levels were deduced from the previously published chromosome counts for these species and/or through comparisons with the relative genome sizes of their closest relatives, for which the chromosome numbers were determined in this study. The relative genome size was measured based on the fluorescence intensity of nuclei stained with the AT-selective fluorochrome 4′,6-diamidino-2-phenylindole (DAPI). The relative genome size (relative 2C value) of a sample was computed as the ratio between the mean G0/G1 peak value of the sample and the mean G0/G1 peak value of the standard with a known genome size. A monoploid relative genome size (relative C*x* value) was also inferred, representing the relative DNA content of a monoploid, unreplicated genome that comprises one set of chromosomes and corresponds to the base chromosome number (*x*) of the taxon (Greilhuber et al., 2005). One of three internal standards was selected for each individual measurement, depending on the genome size of the measured species. *Lycopersicon esculentum* ‘Stupické polní rané’ (2C = 1.96 pg; Doležel et al., 1992) was used as the primary standard for most taxa. In cases when *Lycopersicon* was not suitable (due to overlapping G0/G1 or G2 peaks between the sample and the standard), we used secondary standards, either *Bellis perennis* L. (2C = 3.38 pg; Schönswetter et al., 2007) or *Solanum pseudocapsicum* L. (2C = 2.59 pg; Temsch et al., 2010). The relative genome sizes of all samples that were measured using secondary standards were eventually recalculated relative to the primary standard to obtain a final value for the relative genome size (in arbitrary units), as reported in Section “Results.” The recalculation was performed based on the ratio between the relative genome sizes of the primary and secondary standards. This ratio was calculated based on repeated simultaneous measurements of the primary standard with each secondary standard. In addition to individual sample measurements, selected samples from two or three different species that showed divergent genome size values at the same ploidy level (see section “Results”) were analyzed simultaneously. These measurements were performed to prove and illustrate the differences in genome size between species. All analyses were performed using a Partec Cyflow ML instrument equipped with an HBO-100 mercury arc lamp (Partec, Münster, Germany) following the protocol described in Španiel et al. (2011). The relative genome sizes of the perennials *A. repens* and *A. montanum* were known and taken from previous studies (Španiel et al., 2018; Melichárková et al., 2019), used here in the analysis exploring phylogenetic patterns of relative genome size variation (see below).

### PCR Amplification, Cloning, and Sequencing

Genomic DNA was isolated using the DNeasy 96 Plant Kit, according to the manufacturer’s protocol (Qiagen, Hilden, Germany). One chloroplast and two nuclear DNA regions were amplified and sequenced: the *rpoB*-*trnC*^GCA^ intergenic spacer of chloroplast DNA (cpDNA), the multicopy internal transcribed spacer (ITS) region of rDNA (ITS1-5.8S-ITS2), and the single-copy *DET1* (de-etiolated 1) nuclear gene. Universal primers were used to amplify the *rpoB-trnC* spacer (primers rpoB and trnC^GCA^R; Shaw et al., 2005) and the ITS region (primers ITS4 and ITS5; White et al., 1990). The partial sequence of the *DET1* gene was amplified with the primer set published by Kuittinen et al. (2002), which binds to exons 5 and 8 in *Arabidopsis thaliana* and has previously been shown to perform well also in *Alyssum* (Melichárková et al., 2017). Polymerase chain reaction (PCR) amplifications were performed in a total volume of 20 μl containing 1 μl of gDNA, 0.58 U *Pfu* DNA polymerase (Thermo Fisher Scientific Inc., Waltham, MA, United States), 1× *Pfu* buffer with MgSO_4_, 0.2 mM dNTPs, and 0.2 μM each forward and reverse primers. The *DET1* reaction mix also included 5% dimethyl sulfoxide (DMSO) to reduce possible recombination events during PCR. The following PCR conditions were used: 80°C for 5 min, 30 × (95°C for 1 min, 50°C for 1 min, followed by a 5% ramp to 65°C, 65°C for 4 min), 65°C for 5 min for the *rpoB-trnC* amplification; 94°C for 3 min, 35 × (94°C for 30 s, 50°C for 30 s, 72°C for 1 min), 72°C for 10 min for the ITS amplification; 94°C for 5 min, 30 × (94°C for 30 s, 51°C for 30 s, 72°C for 2 min), 72°C for 7 min for the *DET1* amplification.

PCR products were purified either enzymatically (prior to direct sequencing) using a mixture of Exonuclease I and FastAP Thermosensitive Alkaline Phosphatase (Thermo Fisher Scientific Inc.) or with NucleoSpin Gel and PCR Clean-up column kit (Macherey-Nagel, Düren, Germany), when subjected to cloning before sequencing. ITS products from all individuals and *DET1* products from all diploids were first sequenced directly, and if sequence polymorphisms (double peaks or unreadable parts) were detected in the electropherograms, the PCR products were cloned. *DET1* products from the polyploid individuals were all cloned prior to sequencing. Purified PCR products were cloned with the CloneJET PCR Cloning kit using the pJET1.2/blunt vector, following the manufacturer’s protocol (Thermo Fisher Scientific), with transformation into JM109 competent cells (Promega, Madison, United States). Direct PCR from bacterial colonies was performed in a total volume of 13 μl, using 0.2 μM pJET1.2 sequencing primers, 0.65 U of DreamTaq DNA polymerase (Thermo Fisher Scientific), 1× DreamTaq Buffer with MgCl_2_, and 0.2 mM dNTPs. The cells were lysed by 95°C for 3 min, followed by 35 cycles (95°C for 30 s, 50°C for 30 s and 72°C for 2 min), and a 10-min final incubation step at 72°C. The PCR products were purified by enzymatic clean-up (as specified above) prior to sequencing. Multiple clones per sample were sequenced in an effort to recover all alleles and identify any potential PCR-mediated recombinations: six to eight clones per diploid, 16 clones per tetraploid, and 20 clones per hexaploid sample. Sequencing was performed using the ABI 3730xl DNA analyzer at Eurofins Genomics Company (Konstanz, Germany). The sequences were edited and aligned using Geneious software R7.1.9 (Biomatters Ltd., Auckland, New Zealand) and submitted to the GenBank nucleotide database (Supplementary Table 1). Indels present in the sequence alignments were coded as binary characters (except for those occurring in highly variable homopolymeric stretches that were ignored) according to the simple indel coding approach (Simmons and Ochoterena, 2000) using FastGap 1.2 software (Borchsenius, 2009). The indel coding datasets were appended to the nucleotide datasets and were included in the phylogenetic analyses. The final ITS and *rpoB-trnC* alignments contained sequence data from 152 individuals (76 populations); the *DET1* alignment comprised data from 73 individuals (from a selection of 38 populations; Table 3 and Supplementary Table 1).

**TABLE 3:** Characteristics of the nucleotide alignments used in the present study.

Locus – partition	Alignment length	Number of					Evolutionary model
		Individuals	Sequences	Alleles^*b*^	PI sites^*c*^	Indels	
*rpoB-trnC*	1,013 bp	152	152	61	94 (9.28%)		
*- rpoB* gene	−12 bp				2 (16.67%)		JC
- spacer	−1,001 bp				92 (9.19%)	10	TIM1 + G
ITS rDNA	676 bp	152	265	131	136 (20.12%)		
- 5.8S^*a*^	−190 bp				3 (1.58%)		TrNef + I
- ITS1 + ITS2	−486 bp				133 (27.37%)	3	TIM2 + I+G
*DET1*	603 bp	73	154	75	163 (27.03%)		
- exons	−293 bp				50 (17.06%)		TIM2 + G
- introns	−309 bp				113 (36.57%)	3	GTR + G

*^*a*^Including short fragments of the 18S and 28S genes flanking the ITS1 and ITS2 regions. ^*b*^The total number of different haplotypes (ribotypes, alleles) identified in the alignment. ^*c*^The number of parsimony informative (PI) sites is given in absolute numbers and as percentages of the aligned nucleotide positions. Outgroup sequences are not included in this summary data.*

### Phylogenetic Analyses of the *rpoB-trnC*, ITS, and *DET1* Sequences

The statistical parsimony-based TCS method (Clement et al., 2000) in PopART (Leigh and Bryant, 2015) was used for the identification of different alleles and haplotypes within the datasets, and to determine their frequencies and sharing patterns. A NeighborNet (NN) network (Huson and Bryant, 2006) was generated in SplitsTree4 v. 4.14.4 based on uncorrected P-distances. Phylogenetic trees were inferred using maximum-likelihood (ML, GARLI v.2.01; Zwickl, 2006) and Bayesian analyses (MrBayes v.3.2.6, Huelsenbeck and Ronquist, 2001), both of which were run at the CIPRES Science Gateway (Miller et al., 2010). The three studied DNA markers were analyzed separately, and three data partitions were defined within each dataset as follows: intergenic spacer of *rpoB-trnC*, a fragment of the *rpoB* gene, and indels within the *rpoB-trnC* dataset; non-coding ITS1 and ITS2, 5.8S of rDNA, and indels within the ITS dataset; introns, exons, and indels within the *DET1* dataset. Best-fit models of nucleotide substitutions were assessed in jModelTest v.2.1.10 (Darriba et al., 2012) separately for each nucleotide data partition, using the Akaike information criterion (AIC; Akaike, 1974). Bayesian analyses were conducted with four MCMC (Markov chain Monte Carlo algorithm) chains for 10–15 million generations, with a sampling frequency of every 100th generation. The first 10% of trees were discarded as burn-in and a consensus tree was generated from the remaining trees, computing also the Bayesian posterior probabilities (BPP) for each node. For ML analyses, multiple searches were performed with random starting trees and by setting the program to stop after 20,000 generations if no improvement of the log-likelihood was detected (≤0.01), with a maximum of 215 million generations. Branch support was assessed by 2,000 bootstrap replicates (bootstrap support, BS).

### Inference of Coalescent-Based Species Trees

A Bayesian coalescent-based approach was employed to estimate a species tree using STACEY package v. 1.2.2 (Jones, 2017) for BEAST v. 2.5.1 (Drummond and Rambaut, 2007). Since the multispecies coalescent accounts for incomplete lineage sorting as the primary source of gene tree discordance but not for hybridization events, we assembled two datasets avoiding reticulate evolutionary patterns in the markers and samples (especially in the case of *DET1* data for polyploids, see section “Results”). The first dataset included the *rpoB-trnC* and ITS sequence data, obtained from both diploids and polyploids, whereas the second dataset included all three regions, *rpoB-trnC*, ITS, and *DET1*, from diploids only. The purpose of performing these analyses was to infer species relationships at the diploid level, as well as, to a limited extent, with polyploids involved and confront them with the multilabelled *DET1* gene tree reconstruction. Two populations of diploid *A. fulvescens* sampled from different islands (Samos and Chios islands) were *a priori* defined as two ‘species’, because they were resolved in two divergent clades in each of the gene trees. The tetraploid *A. simulans* was excluded from the analyses because it displayed two divergent ITS copy variants, indicating an allopolyploid origin. All other species (both diploids and polyploids) appeared genetically coherent, without any indication of reticulation or polyphyletic origins when considering only the ITS and *rpoB-trnC* gene trees.

The tool BEAUti 2 (Bouckaert et al., 2014) was used to create a STACEY input file using the following settings and parameters: multiple unlinked data partitions (as defined above), the best-fit nucleotide substitution models, as determined with jModelTest (see above), lognormal relaxed clock, Yule species tree model, and lognormal priors. We ran three independent Markov chain Monte Carlo (MCMC) analyses, with 75 million generations each and a sampling frequency of 10,000 trees, using BEAST v. 2.5.1 through the CIPRES Science Gateway. The BEAST output was analyzed in Tracer v.1.7.1 (Rambaut et al., 2018) and verified for MCMC convergence and the ESS (effective sample sizes) values of parameters. Multiple runs were combined using LogCombiner v.2.5.2, after discarding the first 20% of the sampled trees as burn-in. A maximum clade credibility (MCC) tree was obtained in TreeAnnotator v.2.5.2 and visualized in FigTree v.1.4.4.

To explore the presence of a phylogenetic signal in the measured genome size values of the studied species (described above in section “Chromosome Counting and Flow Cytometry”), we calculated the tree transformation statistics, λ (Pagel, 1999), which estimates the statistical (in)dependence of trait (genome size) evolution and the reconstructed phylogeny. It ranges from 0 to 1, with values close to 0 indicating phylogenetic independence and values close to 1 indicating a phylogenetic signal in the examined trait. It was computed in BayesTraits v.3.0.2 (Pagel et al., 2004) on the combined post-burn-in set of 18,000 trees generated from the three BEAST runs used for MCC tree as described above (the dataset including both diploids and polyploids), with two independent MCMC runs each of 1 million generations and 100,000 burn-in. Since the two runs gave virtually the same results, the posterior distribution of λ values resulting from one MCMC run was depicted in a histogram.

Phylogenetic inference based on the concatenation of the three sequence alignments was discarded in the present study, as this approach can lead to biased topologies with false support, especially in recently diverged lineages (Kubatko and Degnan, 2007). The concatenation was also technically unfeasible, as it is generally not possible to know *a priori* which allele (*DET1*) should be concatenated with which ribotype (ITS) when multiple sequence variants are revealed even in diploid accessions (see Results).

### Estimation of Divergence Times Based on ITS Sequence Data

To estimate the divergence times of the studied species, we assembled an additional ITS alignment with a broader, tribe-wide taxonomic coverage. This alignment followed the dataset compiled by Salmerón-Sánchez et al. (2018), which comprised representatives of almost all genera of the tribe Alysseae (Rešetnik et al., 2013; Španiel et al., 2015), complemented by the ITS sequences of annual *Alyssum* species generated in the present study and the ITS dataset of the perennial *A. montanum–A. repens* species complex (generated in Melichárková et al., 2019). For the tree calibration, we applied an approach described by Huang et al. (2020), who reconstructed a dated phylogeny of the Rosidae clade based on complete plastid sequences and multiple reliable non-Brassicales fossil records and, subsequently, inferred three secondary calibration points within Brassicaceae. These secondary calibrations were used as temporal anchor points for the molecular dating analyses of Brassicaceae tribes based on ITS sequences, representing major lineage splits within the family. Following this approach, the ITS sequences of two outgroup species (*A. thaliana*, tribe Camelineae, lineage I; and *Clausia aprica*, tribe Dontostemoneae, lineage III) were included in the assembled Alysseae dataset to define the ages of the respective nodes (node B representing the split between lineage I and Alysseae; node C representing the split between lineage III and Alysseae, following Huang et al., 2020). In total, the ITS alignment used in the present study comprised 702 sequences from 123 species and 25 genera (Supplementary Table 2). The tree topology and divergence times were estimated using the Bayesian MCMC approach, as implemented in BEAST v. 2.6.1 and run through the CIPRES Science Gateway (Miller et al., 2010). The input file was generated using the tool BEAUti 2. For the tree priors, we selected the general time-reversible (GTR + G) substitution model, the uncorrelated relaxed lognormal clock model, the birth-dead speciation model, and we set the ages of the two nodes with a normal distribution prior, as described by Huang et al. (2020). MCMC was run with 100 million generations and a sampling frequency of 10,000 trees. The BEAST output was analyzed in Tracer v.1.7.1 and checked for MCMC convergence and the ESS values of parameters. An MCC tree was obtained in TreeAnnotator v.2.6.0, discarding the first 25% of the sampled trees as burn-in, and visualized in FigTree v.1.4.4.

## Results

### Chromosome Numbers

**FIGURE 2:**
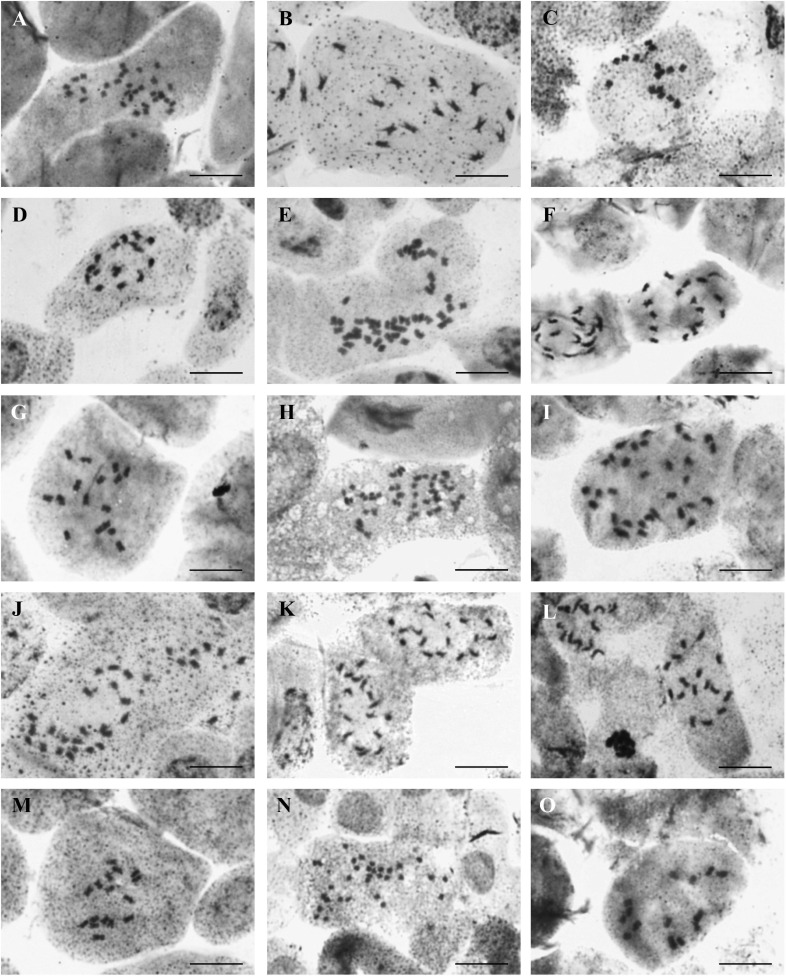
Photographs of chromosome metaphase plates from **(A)** *Alyssum alyssoides* (427AMB-*al*), 2*n* = 32; **(B)** *Alyssum foliosum* (151KAT-*fo*), 2*n* = 16; **(C)** *Alyssum fulvescens* (594PEO-*fu*), 2*n* = 16; **(D)** *A. fulvescens* (595KEK-*fu*), 2*n* = 16; **(E)** *Alyssum hirsutum* (506DRK-*hi*), 2*n* = 48; **(F)** *Alyssum minutum* (319LOM-*mi*), 2*n* = 16; **(G)** *Alyssum simplex* (551RBS-*sx*), 2*n* = 16; **(H)** *Alyssum siculum* (549QUA-*sc*), 2*n* = 48; **(I)** *Alyssum collinum* (261UXA-*cl*), 2*n* = 32; **(J)** *Alyssum simulans* (481PTK-*ss*), 2*n* = 32; **(K)** *Alyssum smyrnaeum* (544DTI-*sy*), 2*n* = 16; **(L)** *Alyssum strigosum* (457SVU-*st*), 2*n* = 16; **(M)** *A. strigosum* (477VAD-*st*), 2*n* = 16; **(N)** *Alyssum turkestanicum* (429ORT-*t*), 2*n* = 32; and **(O)** *Alyssum umbellatum* (458PSS-*um*), 2*n* = 14. Scale bar = 10 μm.

Chromosome numbers were determined in 15 populations of 13 annual species (Supplementary Table 1, Table 2, and Figure 2). For the remaining four species (*A. granatense*, *A. pogonocarpum*, *A. szovitsianum*, and *A. xiphocarpum*), the seeds obtained from the studied populations did not germinate (were unripe or too old) and, therefore, were unusable for chromosome counting. Except for *A. xiphocarpum*, the chromosome numbers of these species were already known from the previously published records (see Table 2). Four different chromosome numbers were detected, 2*n* = 14, 16, 32, and 48, with the base chromosome number *x* = 8 or, exceptionally, *x* = 7 in *A. umbellatum*. They represented the diploid, tetraploid and hexaploid levels.

### Ploidy Level and Genome Size Variation

**FIGURE 3:**
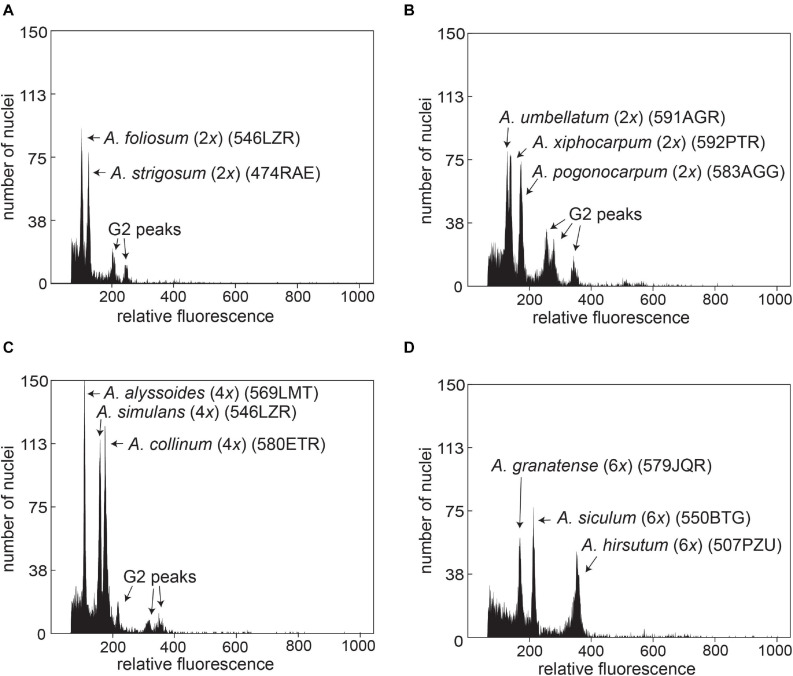
Simultaneous FCM analyses of *Alyssum* species, illustrating the differences in relative genome size values (relative fluorescence) between selected diploid **(A,B)**, tetraploid **(C)**, and hexaploid **(D)** species. For population codes, see Supplementary Table 1.

The relative genome sizes were measured for all sampled 17 annual species (Table 2 and Supplementary Table 1). Both the chromosome number data and genome size values demonstrated uniform ploidy levels within individual species. The chromosome number and ploidy level of *A. xiphocarpum* were previously unknown; therefore, the diploid level inferred here represents the first ploidy record for this species. We also found that the studied annual species showed significant differences in their monoploid genome sizes, exhibiting approximately 2.9-fold variation (Table 2). Differences in genome sizes between several species (and groups within *A. fulvescens*) within the same ploidy level were large enough to yield separate peaks in the FCM histograms during simultaneous analyses, as shown in Figure 3. This variation among species also demonstrated some phylogenetic patterns (see below section “Coalescent-Based Species Trees” for details). The largest relative monoploid genome sizes were detected in *A. hirsutum*, *A. umbellatum*, *A. xiphocarpum*, and, especially, *A. pogonocarpum* (the chromosomes, unfortunately, could not be counted in this species; however, previous records reported the diploid level for this species). In contrast, the smallest relative C*x* values were revealed in *A. alyssoides*, *A. siculum*, *A. turkestanicum*, and *A. granatense* (Table 2).

### DNA Sequence Data Variation

The detailed characteristics of the *rpoB-trnC*, ITS, and *DET1* alignments, such as the alignment length, number of sequences, alleles, polymorphic sites, and indels, are summarized in Table 3.

The *rpoB-trnC* dataset comprised 152 sequences (individuals) with 61 different haplotypes. Haplotype sharing most commonly occurred within populations, as well as among different populations of the same species. In two cases, haplotype sharing was observed between different species: between the tetraploid *A. alyssoides* and the hexaploid *A. siculum*, and between the diploid *A. foliosum* and the tetraploid *A. simulans*. The analyzed species displayed one to six different haplotypes, and the most diverse species were the diploid *A. simplex* and the hexaploids *A. granatense* and *A. siculum*.

Molecular cloning revealed multiple ITS copy variants (ribotypes) within several accessions, in both diploids and polyploids (Supplementary Table 1). Up to eight different ribotypes were, for instance, found in one individual of the hexaploid *A. granatense*; and up to five ribotypes in one individual of the diploid *A. fulvescens*. Altogether, the final ITS alignment comprised 265 sequences retrieved from 152

individuals, which represented 131 different ribotypes. The ribotype sharing patterns were similar to those observed in the *rpoB-trnC* dataset. The same ribotypes were frequently revealed within populations and among different populations of the same species. Ribotype sharing between different species was observed only in two cases: between the tetraploid *A. alyssoides* and the hexaploid *A. siculum*, and between the diploid *A. minutum* and the tetraploid *A. simulans*. The analyzed species displayed one to 15 different ribotypes; the most diverse species were the diploid *A. fulvescens* (12 ribotypes) and the polyploids *A. granatense* (15 ribotypes), and *A. alyssoides* (11 ribotypes).

The *DET1* alignment included four exons and introns among them (although the first intron was missing in several accessions, which was also observed for some perennials by Melichárková et al., 2017). All diploid accessions, except for *A. fulvescens*, appeared homozygous. Multiple alleles were revealed by cloning in the polyploids, with up to six alleles in the hexaploids (see Supplementary Table 1). Altogether, the alignment comprised 154 sequences from 73 individuals, which represented 75 different alleles. No alleles were shared between different diploid species, but allele sharing occurred in several cases between diploids and polyploids (reflecting progenitor-polyploid derivative relationships, see below), and between different polyploid species [those with presumably shared progenitor(s)].

### Phylogenetic Analyses of the *rpoB-trnC*, ITS, and *DET1* Sequences

**FIGURE 4:**
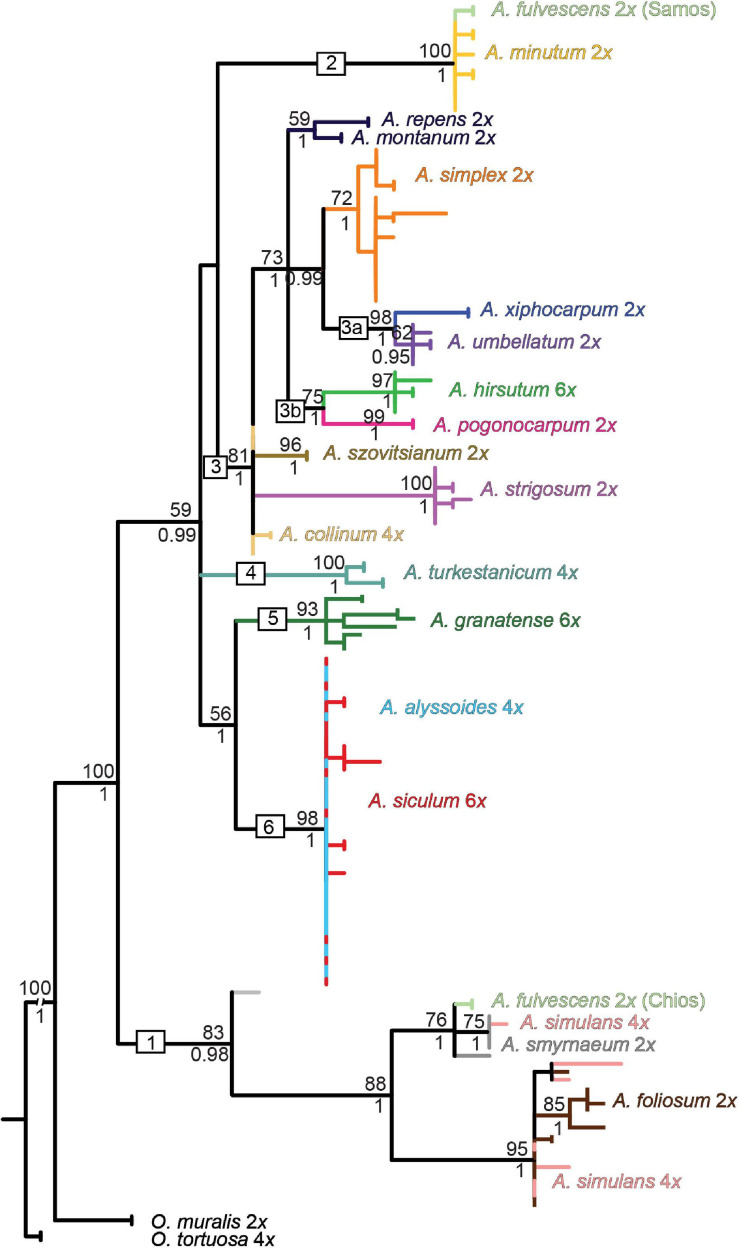
Maximum-likelihood tree based on the *rpoB-trnC* sequences of cpDNA for the studied *Alyssum* species. The values above the branches are bootstrap support (BS) ≥ 50%; those below the branches are Bayesian posterior probability (BPP) values ≥ 0.75 taken from the Bayesian majority-rule consensus tree (not shown). BS and BPP are indicated only for major and species-specific clades, whereas they are skipped for the most terminal clades. Branches and clades are colored according to the species assignment, omitting the terminal labels of individual sequences for the sake of readability (see Supplementary Figure 1A for the fully labeled version of the tree). Each species name is followed by its ploidy level. The geographic origins of the two populations of diploid *A. fulvescens* that exhibited genetic divergence in each of the three DNA regions are indicated (Samos and Chios islands). The numbers 1-6 in boxes denote the clades, as described in the text.

**FIGURE 5:**
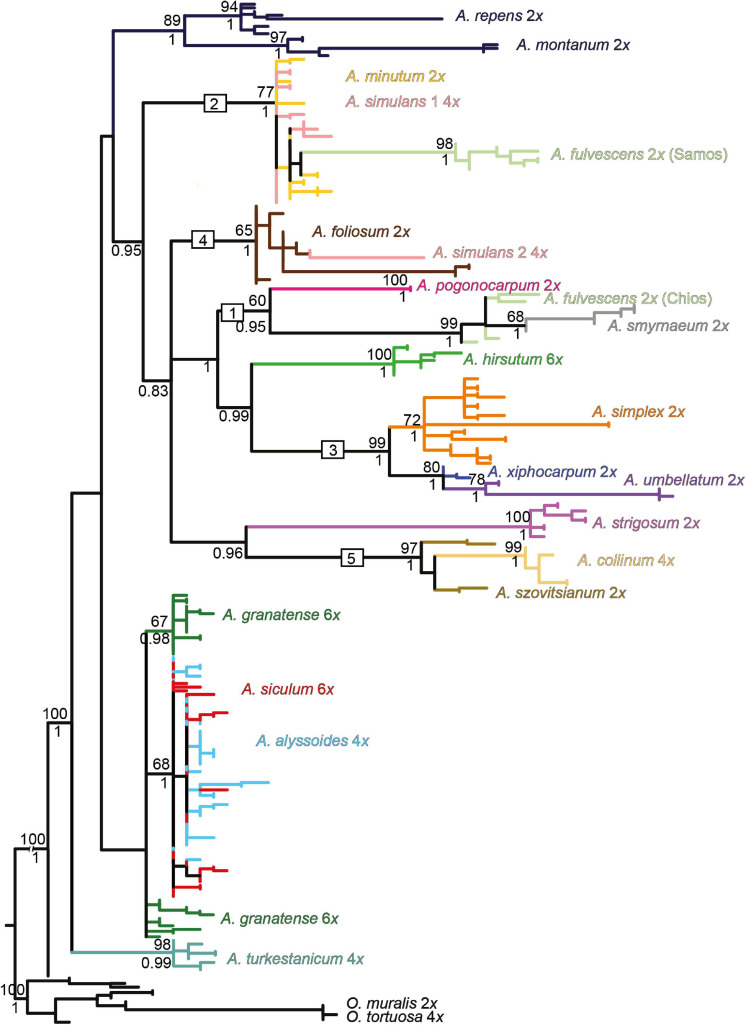
Maximum-likelihood tree based on the ITS sequences of nrDNA for the studied *Alyssum* species. For the tree description, see the legend of Figure 4. Divergent ITS sequences were observed in the tetraploid *A. simulans*, which are placed in distinct clades (nr. 2 and 4), marked here as *A. simulans* 1 and *A. simulans* 2. The fully labeled version of the tree is shown in Supplementary Figure 2A.

**FIGURE 6:**
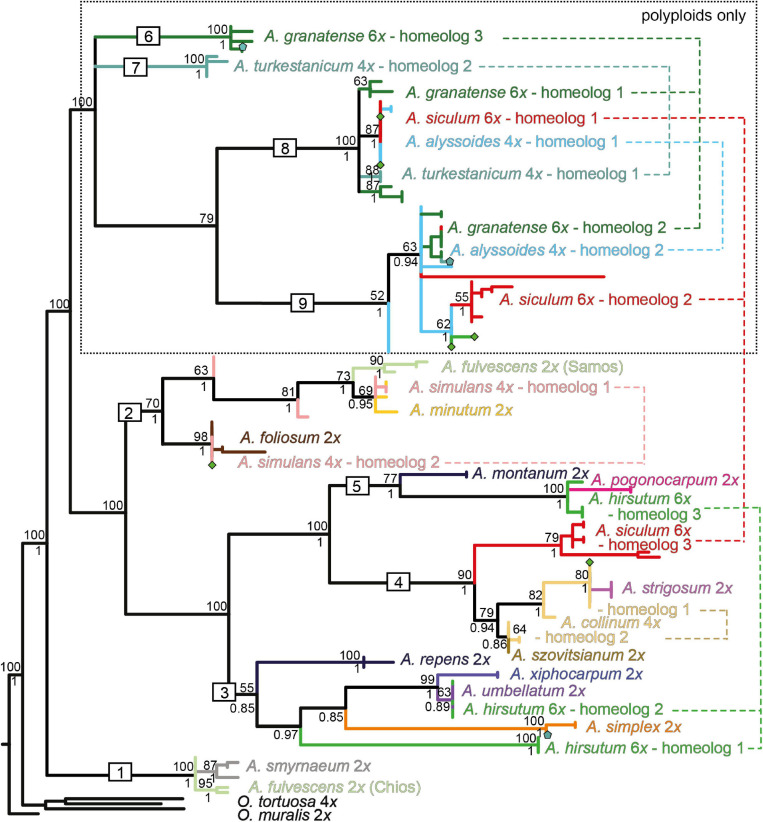
Maximum-likelihood tree based on the *DET1* sequence data for the studied *Alyssum* species. For the tree description, see the legend of Figure 4. The different homeologs identified in the polyploid species are numbered and connected by dashed lines. Symbols (pentagons and diamonds) show the alleles that deviated from the observed homeolog variation in *A. turkestanicum* and *A. hirsutum*, respectively. The fully labeled version of the tree is shown in Supplementary Figure 3A.

The NN networks, the ML, and Bayesian phylogenetic trees exhibited largely congruent topologies for each of the three markers used. Here we present the ML trees (Figures 4–6) with both the bootstrap support (BS) and the Bayesian posterior probability (BPP) values plotted on them. Only the clades that were supported by BS (≥50%) and BPP (≥0.85) are described and interpreted. The NN networks are shown in the Supplementary Figures 1–3.

The ML tree based on the chloroplast *rpoB-trnC* data (Figure 4) showed a relatively well-resolved structure, in which most of the species harbored monophyletic sequences (but see *A. fulvescens*), and several clades could be highlighted. One clade (denoted as clade 1) was composed of four Aegean (sub-)endemics, the diploids *A. foliosum*, *A. fulvescens* from Chios island (594PEO population) and *A. smyrnaeum*, and the tetraploid *A. simulans*. In contrast, *A. fulvescens* from Samos island (595KEK) was placed in another clade together with the more widespread *A. minutum* (clade 2). *A. xiphocarpum* endemic to Lesvos formed a clade with the more widespread Balkan to western Asian *A. umbellatum* (clade 3a). As for the polyploids, the widespread hexaploid *A. hirsutum* was sister to the Aegean diploid subendemic *A. pogonocarpum* (clade 3b). The other four polyploids, *A. turkestanicum, A. granatense, A. alyssoides*, and *A. siculum* (clades 4–6) were clearly differentiated from all here analyzed diploids. While the first two polyploids formed two distinct clades (clades 4 and 5), the last two polyploids appeared completely intermingled (clade 6).

The ML tree based on the ITS data displayed poorly supported backbone relationships (Figure 5), therefore, here we focus only on some well supported patterns. Multiple ITS copy variants obtained from several individuals by cloning were phylogenetically close even in polyploids, placed within the same clades (with a single exception of tetraploid *A. simulans*, see below). Two Aegean diploid subendemics, *A. fulvescens* from Chios island and *A. smyrnaeum*, formed a well-supported clade in a sister position to another Aegean subendemic, *A. pogonocarpum* (clade 1). *A. fulvescens* from Samos island, in congruence with cpDNA data, was placed in a different clade together with *A*. *minutum* (clade 2). *A. xiphocarpum* endemic to Lesvos together with *A. umbellatum* were resolved in a clade with the more widespread *A. simplex* (clade 3). As for the polyploids, clearly polyphyletic ITS sequences were observed only in tetraploid *A. simulans*, placed intermingled either in the clade with *A. minutum* (clade 2) or in the clade with *A. foliosum* (clade 4), the latter position being in congruence with the *rpoB-trnC* data. The tetraploid *Alyssum collinum*, so far reported from southwestern Europe and northern Africa, was placed with much support within the clade of Asian *A. szovitsianum* (clade 5). Four polyploids, *A. turkestanicum, A. granatense, A. alyssoides*, and *A. siculum*, similarly as in the *rpoB-trnC* data, appeared differentiated from all here analyzed diploids, but only *A. turkestanicum* formed a separate well-supported clade. The NN network (Supplementary Figure 2B) displayed a structure that supported the topology of the ML tree, but, in addition, indicated conflicting, reticulated patterns for two polyploids: the tetraploid *A. simulans* against the clades 2 and 4; and the hexaploid *A. hirsutum* against the clades 1 and 3.

The ML tree based on the *DET1* data (Figure 6) showed low resolution at the backbone (in congruence with the NN network structure, see Supplementary Figure 3B), but more terminal clades and the relationships among them generally received high support. The diploid species were placed in five distinct clades (clades 1–5), in agreement with the splits resolved in the NN network. In congruence with the ITS data, two Aegean subendemics, *A. fulvescens* from Chios island and *A. smyrnaeum*, formed one clade (clade 1), whereas *A. fulvescens* from Samos island was sister to *A. minutum* (within clade 2). *A. xiphocarpum* endemic to Lesvos together with *A. umbellatum* were resolved in a clade with the more widespread *A. simplex* (clade 3). Finally, the remaining three annual diploids, *A. pogonocarpum, A. strigosum*, and *A. szovitsianum*, were placed within the clades 4 and 5. The sequences obtained from the polyploids were scattered across the tree, placed within four of the five above-mentioned clades of the diploids, plus in four additional clades (clades 6–9) comprising only polyploids. Within each polyploid, we revealed divergent alleles, which were placed in separate clades of the trees, indicating the presence of two or three different homeologs, i.e., gene copies derived from the parental species (Figure 6). In four polyploids, *A. alyssoides, A. granatense, A. siculum*, and *A. turkestanicum*, we observed two to three divergent homeologs, which were placed in the clades comprising exclusively polyploids, i.e., not matching any of the analyzed diploids.

### Coalescent-Based Species Trees

**FIGURE 7:**
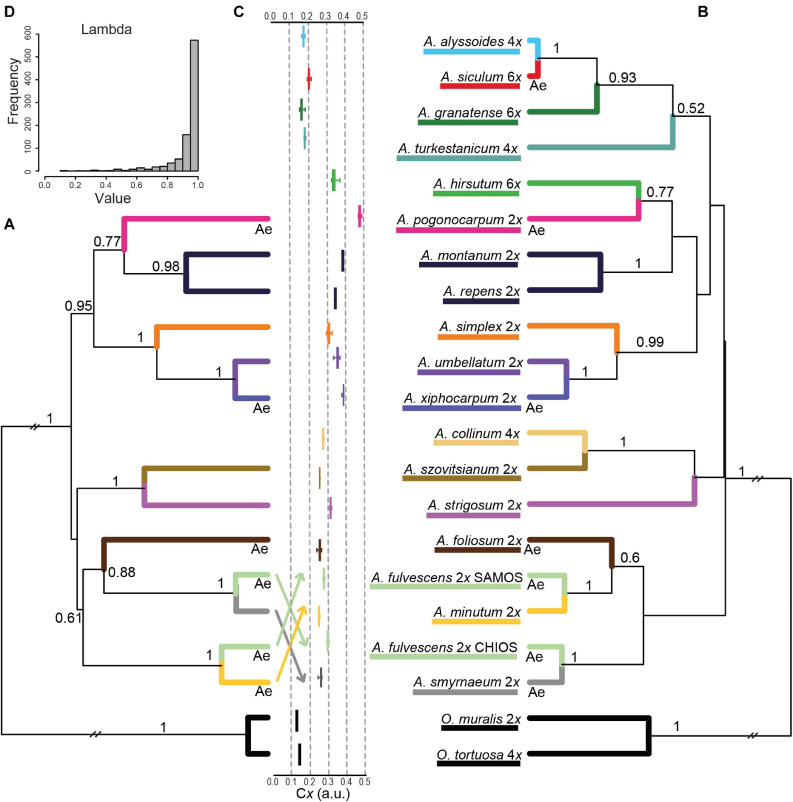
Results of the species tree inference in the studied *Alyssum* species. The maximum clade credibility species trees, which were obtained from the coalescent analysis in BEAST are shown, based on **(A)** cpDNA, ITS, and *DET1* datasets for diploids, and **(B)** cpDNA and ITS datasets for both diploids and polyploids (excluding the tetraploid *A. simulans*, see section “Materials and Methods”). Posterior probability values ≥ 0.5 are indicated above the branches. Ploidy levels are shown next to the species names. ‘Ae’ below branches indicates the placements of Aegean subendemics referred to in the text. **(C)** Boxplots depicting the relative monoploid genome size (C*x*) values measured for the studied species. See Table 2 and Supplementary Table 1 for the precise population- and species-level C*x* values. **(D)** Posterior distribution of the tree transformation statistic λ (Pagel, 1999), computed by MCMC analyses in BayesTraits, quantifying the phylogenetic signal in genome size variation.

The species tree inferred from all three DNA markers of diploids is depicted in Figure 7A. It includes one larger clade (BPP = 0.95) comprising two Aegean (sub)endemics (*A. pogonocarpum* and *A. xiphocarpum*) and two species with wider ranges (*A. simplex* and *A. umbellatum*), together with the nested *A. montanum* and *A. repens* perennials. The rest of the tree displayed low resolution at higher-level relationships, and only smaller clades can be highlighted as follows. The widespread southern Europe-southwestern Asian *A. strigosum* formed a clade with southwestern Asian *A. szovitsianum* (BPP = 1). In agreement with the individual gene trees, *A. fulvescens* from Samos was resolved in the sister position to widespread *A. minutum* (BPP = 1), whereas *A. fulvescens* from Chios was sister to the Aegean subendemic *A. smyrnaeum* (BPP = 1). Another Aegean subendemic, *A. foliosum*, clustered with the latter two species, albeit with somewhat lower support (BPP = 0.88). The species tree based on cpDNA and ITS alignments and including both diploids and polyploids (Figure 7B) showed virtually the same topology as the former species tree (in terms of the relationships among the diploids), but with lower BPP for some clades. Two polyploids, *A. hirsutum* and *A. collinum*, were placed within the clades of diploids. In contrast, the polyploids *A. granatense, A. siculum*, and *A. alyssoides* formed a distinct clade (BPP = 0.93), and together with another polyploid *A. turkestanicum*, were separated from the diploids.

The boxplots showing the relative monoploid genome size values (C*x*) measured in the studied species (Figure 7C) and plotted next to the species tree indicate that the observed genome size variation is phylogenetically structured. The presence of a strong phylogenetic signal in the genome size data was, indeed, supported by the posterior distribution of λ values, which were skewed toward 1 (Figure 7D).

### Estimation of Divergence Times Based on ITS Sequence Data

The dated phylogeny based on the ITS sequence data (Supplementary Figure 4) suggested that the most recent common ancestor (MRCA) of the genus *Alyssum* evolved during the Tortonian Age of the Late Miocene epoch [median age of 9.58 mya, 95% high posterior density (HPD): 7.11–14.09]. The origin and early diversification of the annual lineage examined in this study, including the nested subclade of the perennial *A. montanum-A. repens* species complex, could be dated back to the Late Miocene (Messinian Age) up to the Early Pliocene (median age of the MRCA: 5.38 mya, 95% HPD: 4.15–7.85). The annuals were split into three clades. The first clade, comprising most of the species, originated and began to diversify as early as in the Early Pliocene (median age of 4.66 mya, 95% HPD: 3.36–6.44), followed by further speciation during the Pliocene and Pleistocene. The MRCA of the two other clades, comprising the remaining four and three species, were inferred to appear later (median ages of 3.51 mya, 95% HPD: 2.59–5.39; and 1.71 mya, 95% HPD: 0.91–3.47, respectively). In summary, when confronted with the clades resolved in the species tree (Figure 7A, see also Table 4), we can derive that the annual species examined in this study have likely originated in the Late Pliocene and the Pleistocene epochs.

**TABLE 4:** Age estimates (median and 95% of the highest posterior density, HPD) in millions of years for the nodes of the given clades of the studied *Alyssum* species, as inferred from the relaxed molecular-clock analysis, performed in BEAST and based on ITS sequence data (see Supplementary Figure 4 and Supplementary Table 2).

Clade	Median	95% HPD lower − upper interval	BPP
*A. smyrneum* + *A. fulvescens* (Chios)	0.85	0.42–1.70	1.00
*A. minutum* + *A. fulvescens* (Samos)	1.71	0.91–3.47	1.00
*A. szovitsianum* + *A. collinum* + *A. strigosum*	3.27	2.15–5.33	0.97
*A. szovitsianum* + *A. collinum*	1.22	0.71–2.33	1.00
*A. umbellatum* + *A. xiphocarpum* + *A. simplex*	1.40	0.89–2.39	1.00
*A. umbellatum* + *A. xiphocarpum*	0.53	0.26–1.14	1.00
*A. alyssoides* + *A. siculum* + *A. granatense* + A. turkestanicum*	3.51	2.59–5.39	1.00
*A. alyssoides* + *A. siculum* + *A. granatense*	1.20	0.77–2.20	1.00
*A. alyssoides* + *A. siculum*	0.68	0.44–1.21	1.00
All studied annual species incl. the perennial *A. montanum–A. repens* species complex	5.38	4.15–7.85	1.00

*The clade on the calibrated ITS tree includes also nested A. montanum-A. repens species group. Only the clades resolved on the coalescence species trees (see Figure 7) are listed here. The BPP column shows posterior probability values for the given clades on the calibrated ITS tree (Supplementary Figure 4).

## Discussion

### Distribution and Diversity Patterns of the Studied *Alyssum* Annuals: An Evolutionary Hotspot in the Aegean Region

The phylogenetic lineage of the *Alyssum* species examined in the present study includes 16 species growing in Europe. Their distribution is centered in southern Europe, especially in the eastern Mediterranean and the Aegean archipelago (Jalas et al., 1996; Hartvig, 2002; Strid, 2016). As many as 12 species occur in the Aegean region, seven of which are subendemics that are either restricted to this region or extend only to the adjacent mainland regions of Greece, North Macedonia, western Turkey or Sicily (Dudley, 1965; Hartvig, 2002; Strid, 2016). Our phylogenetic reconstructions revealed that these Aegean subendemics do not form a single monophyletic group but are placed in several distinct clades across the inferred species trees (Figure 7), and include both diploids and polyploids. Along with the divergence time estimates, these results suggest that the Aegean subendemics likely originated at different times and through different modes. Thus, it seems that the Aegean region has repeatedly acted as a significant evolutionary center favoring multiple speciation events in this phylogenetic lineage. Indeed, this area is recognized to be one of the major biodiversity hotspots of the Mediterranean Basin, featuring high species richness and a significant proportion of narrow endemics (Médail and Quézel, 1997; Panitsa et al., 2018). Several factors have been highlighted that are likely to drive plant diversification and distribution patterns in the Aegean area, especially its complex climatic and geological history, high topographical and geological heterogeneity, which contributes to the formation of large habitat diversity, as well as the long-term presence and influence of humans (Sfenthourakis and Triantis, 2017; Panitsa et al., 2018).

In this study, we have inferred that the origin and early diversification of the studied lineage of annual *Alyssum* species could be dated back to the Late Miocene and Pliocene, between the Messinian salinity crisis (Krijgsman et al., 1999) and the establishment of the Mediterranean climate (Suc, 1984). The onset of the Mediterranean climate was a major climatic change, which likely promoted the further diversification of this lineage, in a similar manner as identified for some other Mediterranean-adapted plant groups (Fiz-Palacios and Valcárcel, 2013; see also Carnicero et al., 2017). Most extant *Alyssum* species, including those that are distributed throughout the Aegean, originated at the end of the Pliocene or during the Pleistocene. This was the era when the climatic oscillations caused sea-level changes and recurrent land connections and disconnections (Perissoratis and Conispoliatis, 2003). They affected the speciation processes by facilitating species dispersal during sea-level drops in glacial periods, and increasing the degree of range fragmentation and population isolation during interglacial and postglacial periods (Nieto Feliner, 2014; Simaiakis et al., 2017; Panitsa et al., 2018). The *Alyssum* species do not bear specific adaptations to facilitate the long-distance dispersal of seeds; instead, field observations suggest that seed dispersal occurs primarily through gravity and zoochory (sheep and goats), and thus also through human activities, mainly cattle farming and grazing. Therefore, geographic distance and barriers may efficiently reduce gene flow, which is manifested by the existence of a few narrow endemics. Interestingly, most species that occur in the Aegean region are largely sympatric (Table 1), with several species recorded within one island (e.g., four species in Lesvos and Samos, and six in Crete), with as many as five species recorded from a single locality in Crete (Supplementary Table 1). Thus, a simple vicariance scenario associated with allopatric differentiation that has generally been favored in the fragmented Mediterranean landscape (Thompson, 2020) and has been observed for a number of Mediterranean plant groups (e.g., Bittkau and Comes, 2009; Crowl et al., 2015), cannot explain the *Alyssum* species diversity observed in the Aegean region. Considering the common co-occurrence of multiple species within a single site, ecological speciation (Coyne and Orr, 2004) does not appear to play a determinant role either, despite the wide range of occupied habitats, the substrate-specificity observed in some cases (Hartvig, 2002; Strid, 2016, see also Table 1), and the large elevational gradient. However, detailed ecological niche characterization and modeling to compare the potential and realized niche spaces of the species (see, e.g., López-Jurado et al., 2019; Castro et al., 2020) will be needed in future to address this issue in detail. We assume that the restricted distribution of some narrow endemics is more likely due to low-dispersal ability rather than environmental constraints [as inferred e.g., by Crowl et al. (2015)].

The distribution ranges can change over time, either naturally or due to human factor, which both can be expected in the biogeographically and geologically complex Aegean area with the long history of human presence (Panitsa et al., 2018; Thompson, 2020). When inspecting the phylogenetic relationships in comparison with the distributional patterns, a specific pattern emerged in some cases. Several diploid species and their closest relatives (also diploids) occur parapatrically or sympatrically, with one species characterized by a restricted distribution (being a narrow endemic), whereas the other occupying a much broader range. This finding was observed for the species pair *A. umbellatum* and *A. xiphocarpum*, as well as for species from the clade comprising *A. fulvescens*, *A. smyrnaeum*, *A. minutum*, and *A. foliosum* (Figure 7). This pattern resembles a specific type of allopatric speciation, which is referred to as founder-event speciation (Templeton, 2008), and was recently inferred in the Mediterranean genus *Cymbalaria* (Carnicero et al., 2017). This mode of speciation involves the establishment of a new (peripheral) population from a larger ancestral population, and its genetic divergence due to genetic drift, as well as divergent selective response (Naciri and Linder, 2020). We propose that such events may have been facilitated by recurrent range shifts, expansion or fragmentation, in response to sea-level changes in the Pleistocene (Nieto Feliner, 2014). In addition, since the studied *Alyssum* annuals commonly grow on disturbed sites and in pastures, human-driven colonization due to cattle transport may also have shaped and changed the distribution patterns. Thus, even though these sister species now grow sympatrically, they may have evolved through allopatric pathways at a finer spatial scale. The development of some premating isolating barriers (Coyne and Orr, 2004) have also likely played an important role in the speciation process, as phenological shifts have been observed among some of these co-occurring, closely related species (personal observations, see also below).

A few recent biogeographic studies that have focused on the Aegean area have investigated the diversification, speciation, and colonization processes of some plant groups in detail (Crowl et al., 2015; Jaros et al., 2018; Carnicero et al., 2021). These studies have suggested primarily allopatric, non-adaptive lineage diversification and speciation, driven by geographic isolation and random drift. These processes have been associated with the formation of the mid-Aegean Trench in the Middle Miocene (12–9 mya), when the ancient Aegean landmass became fragmented (Sfenthourakis and Triantis, 2017), and the Pleistocene-era sea-level oscillations, depending on the age of the studied species (Bittkau and Comes, 2005, 2009; Crowl et al., 2015; Jaros et al., 2018). In other cases, sympatric ecological speciation and founder-event speciation have also been inferred (Carnicero et al., 2017, 2021). Thus, the few detailed studies that have been performed so far, along with the here presented scenarios, highlight the complexity of speciation processes in the Aegean region.

### Multiple Allopolyploid Speciation Events Increased the Species Diversity Across the Whole Distribution Range

We revealed the presence of diploid, tetraploid, and hexaploid levels among the studied *Alyssum* annuals, in accordance with earlier reports (summarized in AlyBase, see Španiel et al., 2015). We did not observe ploidy level variation within populations or within species. The diploids and tetraploids that were traditionally classified within *A. simplex* were recently split into two separate species, *A. simplex* (diploids) and *A. collinum* (tetraploids), which was substantiated by preliminary molecular data, by differences in the monoploid genome size, and some morphological traits (Cetlová et al., 2019). Indeed, the here presented phylogenetic analyses placed these two species in two distinct clades. In addition, the parentage of the tetraploid *A. collinum* did not appear to involve the diploid *A. simplex* (see also below). Some ploidy level variation was previously reported within a few species (see Table 2); however, these observations remain to be confirmed by future studies. In this study, all examined annual polyploids (seven species in total) appeared to evolve through allopolyploidy. Interestingly, only three of these polyploids occur in the Aegean region, thus, it seems that although the polyploidization played a certain evolutionary role in this area as well, it was not the prevalent speciation mechanism there.

**FIGURE 8:**
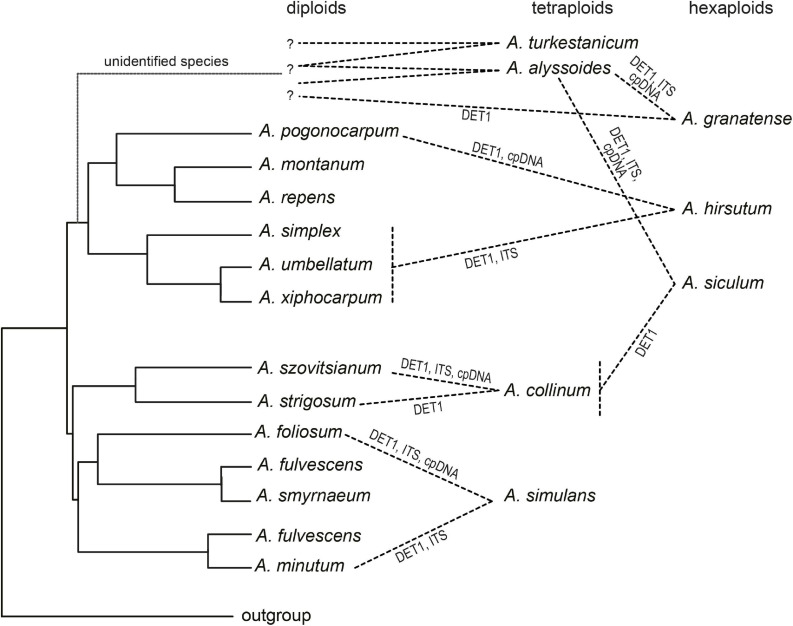
Scheme summarizing the allopolyploidization events in the studied *Alyssum* species, as inferred from DNA sequence data. Diploid species are depicted in the coalescent species tree, as shown in Figure 7A, based on all three markers used, whereas polyploids are connected to their inferred diploid or lower-ploidy progenitors. Question marks indicate unidentified diploid progenitors (species currently not occurring in Europe, possibly of Asian origin or extinct). See the text for further details.

The allopolyploid origins of the studied annuals were derived from the cpDNA–ITS incongruence, from ITS intragenomic polymorphisms, and from single-copy (*DET1*) homeolog variation. The combination of multiple markers demonstrating different patterns of inheritance and molecular evolution has been shown to be the most efficient approach for reconstructing reticulate evolution (Naciri and Linder, 2015; e.g., Záveská et al., 2016; Díaz-Pérez et al., 2018; Mandák et al., 2018). In this study, we combined the nrDNA region that is prone to sequence homogenization by concerted evolution and may show complex patterns in polyploids (Álvarez and Wendel, 2003; Nieto Feliner and Rosselló, 2012), cpDNA with uniparental inheritance, and a biparentally inherited single-copy nuclear gene with the potential to retain parental subgenomes (Small et al., 2004; Nieto Feliner and Rosselló, 2012). In some cases, we were able to identify the most likely parental species or at least the phylogenetic lineages involved in the polyploid origins, which are discussed below and summarized in Figure 8.

The tetraploid *A. simulans*, which is distributed in southeastern mainland Greece, Peloponnese, Evvia, and Crete, was the only species in which divergent, non-homogenized ITS variants were revealed, clustering with two distinct diploids, Aegean subendemic *A. foliosum* and widespread Mediterranean *A. minutum*, in concert with the identified *DET1* homeologs. The former diploid was revealed as the probable maternal parent, based on the cpDNA patterns. All three species occur in sympatry, and these distribution patterns, non-homogenized nrDNA, and almost identical genome size (monoploid C*x* values) suggested a recent, likely Late Pleistocene or even postglacial allopolyploid origin. *Alyssum simulans* was described only recently, and its hybrid origin was already hypothesized based on morphological intermediacy (Hartvig, 2002). It remains uncertain, however, whether *A. simulans* originated multiple times, i.e., independently in Crete and in mainland Greece, or just at a single site and expanded to its current range after its origin.

The hexaploid *A. hirsutum* is widely distributed across eastern Europe, especially in the Black Sea region, extending to the Caucasus region (Grossheim, 1949; Jalas et al., 1996), but does not grow in the Aegean area. One of its parental species was suggested to originate from the clade comprising *A. xiphocarpum* (which is endemic to Lesvos), *A. umbellatum* (Aegean subendemic), and *A. simplex* (throughout the entire Mediterranean, reaching the Caucasus). Thus, either *A. simplex* (being more similar to *A. hirsutum* morphologically than the other two species, which have short umbellate fruiting racemes) or a common ancestor of that clade appear to be the most likely parental species. All three species have similar monoploid genome size values, which are also close to *A. hirsutum*. The identity of the other parental species remains puzzling because both cpDNA and *DET1* point to *A. pogonocarpum*, a species restricted to Rhodos and southwestern Turkey (Kleinsteuber et al., 2016), which might be supported by the morphological resemblance between these species (very long hairs on fruits, Carlström, 1984), but occurs allopatrically and also has a markedly larger genome size (Figure 7C and Table 2). Nevertheless, also an unsampled, related species from the Irano-Anatolian or Caucasus areas could have been involved in the origin of hexaploid *A. hirsutum* (see also below).

The tetraploid *A. collinum* contained ITS and cpDNA sequences that both clustered together with the diploid *A. szovitsianum*. In the *DET1* locus, two slightly divergent groups of alleles were identified, clustering with the sister species *A. szovitsianum* (southwestern Asia) and *A. strigosum* (southern Europe-southwestern Asia). This pattern favors an allopolyploid origin involving progenitors close to these two relatives. Also the monoploid genome size of *A. collinum* is clearly intermediate between these two species (Figure 7C and Table 2). The distribution of *A. collinum* remains poorly understood because it has only recently been taxonomically segregated from the widespread Mediterranean diploid *A. simplex* (Cetlová et al., 2019). The occurrence of *A. collinum* is so far confirmed only from the western Mediterranean (Cetlová et al., 2019, and the present samples), and no records exist from eastern Europe. Some tetraploid individuals (under the name *A. simplex*) were reported from Iran and Tajikistan (see Table 2), but it is unclear if they could be conspecific with *A. collinum* or represent autotetraploids of *A. simplex*, or even another species. Since the present sequence data suggest that the parental species of *A. collinum* came from the eastern regions, it is evident that the taxonomic identity of the Asian tetraploids should be resolved to delimit the distribution range of *A. collinum* completely, and to understand its origin and evolution.

The remaining four polyploids (the tetraploids *A. alyssoides* and *A. turkestanicum*; the hexaploids *A. granatense* and *A. siculum*) did not show closer affinities to any of the European diploids; however, their divergent *DET1* sequences (Figure 6), which were placed in clearly distinct clades, strongly favored independent allopolyploid origins. The most widespread tetraploid *A. alyssoides* displayed two divergent *DET1* copies, which were both present in the hexaploids *A. siculum* and *A. granatense*, and one in the tetraploid *A. turkestanicum*. Thus, *A. alyssoides* was most likely involved in the origin of both hexaploids, i.e., the western Mediterranean *A. granatense* (which is in congruence with their similar morphology, Küpfer and Nieto Feliner, 1993) and the eastern Mediterranean *A. siculum*. The third *DET1* homeolog of *A. granatense*, in agreement with the distinct clade formed by its cpDNA sequences, pointed to another progenitor, which, however, remained unidentified. The hexaploid *A. siculum*, based on previous morphological and molecular data, was suggested to be either an allopolyploid, derived from *A. alyssoides* and *A. simplex*, or an autopolyploid of *A. alyssoides* (Persson, 1971; Rešetnik et al., 2013). In this study, we found that *A. siculum*, which is distributed in Crete, Peloponnese, mainland Greece, and Sicily, indeed, displayed lot of shared variation in all three markers with *A. alyssoides*, which may suggest a very recent polyploid origin. In contrast, we did not find any evidence that *A. simplex* could be the second parent. Instead, one of the *DET1* homeologs revealed in *A. siculum* was sister to the clade containing *A. collinum*, *A. szovitsianum*, and *A. strigosum*, which suggests that one of these species may have been the paternal parent of the hexaploid *A. siculum*. Based on the present records, only *A. strigosum* occurs sympatrically (in mainland Greece as well as Crete; Hartvig, 2002; Strid, 2016) with the hexaploid; however, the highest morphological resemblance points to the southwestern Asian *A. szovitsianum* (personal observation). We did not observe any genetic differentiation between the accessions of *A. siculum* from mainland Greece, Crete, and Sicily that could suggest the independent, polytopic origins of this hexaploid. However, the present analyses were based on only a few loci and may not be sufficient to detect multiple polyploid origins. Where this polyploid originated and how it reached its current disjunct distribution also remains an open question. The sea barriers between mainland Greece, Crete, and Sicily have persisted since the late Miocene; therefore, either a natural long-distance dispersal event occurred or introduction by man was possible, as it commonly grows on disturbed sites and pastures. Finally, the tetraploid *A. turkestanicum*, which is distributed from the Balkan Peninsula to eastern Europe, Black Sea Region, Caucasus, Near East, central Asia, and Siberia (Rechinger, 1968 as *Alyssum desertorum*; Rybinskaya, 1994; Jalas et al., 1996; Zhou et al., 2001; German, 2003; Marhold, 2011), formed a distinct clade according to both the ITS and cpDNA-based trees, with no obvious affinities to any European diploids. One *DET1* homeolog was in a clade with *A. alyssoides*, which suggests several scenarios: *A. alyssoides* may have been involved in its origin, both species may share a common diploid ancestor of extra-European origin, or homeolog sharing may also reflect local hybridization events. Interestingly, these four polyploids (*A. alyssoides, A. turkestanicum, A. granatense*, and *A. siculum*) have the smallest monoploid genome sizes from all here analyzed species (Figure 7C), which may suggest that their unidentified diploid progenitors also have small genomes, and/or that genome-downsizing occurred as a result of diploidization, a process commonly observed in polyploids (Dodsworth et al., 2016). Genome-downsizing, however, was not observed in the other three polyploids (*A. collinum, A. simulans*, and *A. hirsutum*); their genome sizes were rather correlated with the phylogenetic positions and, thus, reflected the genome size variation among their diploid ancestors (Table 2 and Figure 7C).

The significance of recent polyploidization events (neopolyploidy) for the diversification and speciation processes has been inferred for a number of Mediterranean genera, which have been especially linked to climate-induced range shifts and secondary contacts during the Pleistocene (e.g., Cecchi et al., 2013; Frajman et al., 2016; Wagner et al., 2019). Except for two polyploids, *A. siculum* and *A. simulans*, which are both narrowly distributed and restricted to certain parts of the Mediterranean regions (SE Greece, Crete, plus Sicily, in the case of *A. siculum*) and supposedly of very recent origins, the other studied polyploids have broader distributions, some also occurring in extra-Mediterranean areas (e.g., the Caucasus, central Asia). This finding may support the hypothesis that allopolyploids, harboring greater genetic variation, complex and dynamic genomes, could become successful colonizers that exhibit larger distribution areas and different, often wider, ecological niches than diploids (Soltis et al., 2014; see e.g., Arrigo et al., 2016; Cornille et al., 2016). Still, it may be premature to favor or reject this hypothesis with the present data, as in the case of these widespread *Alyssum* polyploids, either at least one of its diploid progenitors also shows widespread occurrence (cases of *A. hirsutum* and *A. granatense*), or the diploid progenitors (and their ecological and chorological characteristics) remain unknown (cases of *A. turkestanicum* and *A. alyssoides*).

### Phylogenetic Patterns, Cryptic Diversity, and Further Perspectives

The phylogenetic trees that were generated based on the three genetic markers employed in this study differed in their resolution. The best resolved hierarchical structure was obtained from the cpDNA tree, in which most species were monophyletic, and the higher-level relationships received at least moderate support. Few species appeared paraphyletic, which most likely reflects lack of resolution or shared ancestral variation, as this pattern was only observed in some closely related diploids (e.g., *A. minutum* and *A. fulvescens* from Samos; *A. smyrnaeum* and *A. fulvescens* from Chios) or indicated the maternal origins of some polyploids (e.g., *A. siculum* and *A. alyssoides*; *A. simulans* and *A. foliosum*). In contrast, both the ITS- and *DET1*-based trees displayed a low resolution in the tree backbone, whereas the more terminal clades of pairs or groups of closely related species were well-supported and largely congruent among all three markers. The low resolution of the nuclear trees, despite the sufficient number of variable sites, most likely mirrors the more complicated evolution of biparentally inherited markers, in which past interspecific gene flow, during and after speciation, polyploidization, recombination, and, in the case of ITS also processes of sequence homogenization, may have obscured the divergence history, and resulted in conflicting, reticulate patterns (Nieto Feliner and Rosselló, 2012; Naciri and Linder, 2015). In agreement with the individual gene trees, the initial diversification patterns were poorly resolved in the species tree, although the terminal clades of the closest relatives received high support. The close relatedness of some species, as was revealed in this study, was already suggested based on their morphological similarity (e.g., *A. fulvescens* and *A. smyrnaeum*; *A. xiphocarpum* and *A. umbellatum*; Hartvig, 2002). An intriguing case remains the eastern Aegean endemic *A. fulvescens* (distributed in Samos, Chios, and two smaller islands of Patmos and Kalymnos, Strid, 2016). The populations from the Chios and Samos islands that were analyzed in this study were genetically differentiated, despite no obvious morphological distinction. Consistently for each genetic marker, the samples from Chios were genetically closest to *A. smyrnaeum* (the more widespread Aegean subendemic, which grows in both Samos and Chios islands, Strid, 2016 and present records), whereas those from Samos clustered with *A. minutum* (a widespread Mediterranean species, including the Aegean region, but not recorded from Samos and Chios). This pattern cannot be explained by a recent hybridization or introgression event with either of these species, considering also the fact that *A. fulvescens* from Samos grows at the same site, together with *A. smyrnaeum* (pop. 595KEK), but they apparently do not hybridize nowadays and they display some phenological shifts (our field observations). Thus, we can only speculate whether the present patterns are due to past hybridization events that caused genetic divergence within *A. fulvescens* or whether they indicate the existence of two independent and separately evolving entities with low levels of morphological differentiation, also known as cryptic species (Fišer et al., 2018; see, e.g., Crowl et al., 2015, 2017; Padilla-García et al., 2018). Considerable among-population variation was observed in genome size values in this species, although it does not correlate precisely with genetic patterns. Still, these patterns may indicate some hidden evolutionary processes in this species. In the future, more detailed investigations of morphological and genetic variation in *A. fulvescens* should be undertaken to solve this issue.

Genome size variation observed among the analyzed species was congruent with the phylogenetic patterns (Figure 7). The monoploid relative genome size of the annual species studied here ranged from 0.161 in *A. granatense* to 0.474 in *A. pogonocarpum* (Table 2). Perennial species of the *A. montanum–A. repens* complex, according to previously published studies, showed a much narrower range of values, between 0.296 ± 0.004 (in some populations of *A. repens*, Melichárková et al., 2019) and 0.389 ± 0.006 (in *Alyssum cacuminum*, Španiel et al., 2018, 2019). These values are within the range of those of the here studied annuals, in agreement with the phylogenetic position of the perennial *A. montanum–A. repens* group, nested within the lineage of annuals, suggesting that these perennials could have evolved from the annuals. It has been proposed and also documented in several cases that annuals tend to have smaller genomes compared to perennials (e.g., Frajman et al., 2015; Zahradníček et al., 2018), albeit this correlation may be also due to the common association of annual life history with higher rates of selfing (Albach and Greilhuber, 2004). Here, we did not support this tendency, in congruence with the findings by Cacho et al. (2021), as genome size variation displayed significant phylogenetic correlation, irrespective of the annual or perennial life history. It has been shown that most of genome size variation in plants is attributable to the differential evolution of repetitive DNA components, which may represent a highly dynamic process that is correlated with species relatedness and phylogeny (McCann et al., 2020).

One limitation of the present study, however, is that we did not include species and populations from the extra-European range (except of *A. szovitsianum*). About 12 species from northern Africa and southwestern to central Asia not sampled here (Dudley, 1964a, 1965), will be needed in future to resolve the evolutionary and biogeographic history of these *Alyssum* annuals completely. So far, up to five extra-European species were included in previous tribus- or genus-wide phylogenetic studies (Li et al., 2015; Huang et al., 2020), which supported their placement within the here studied clade of annuals, and appeared close to some more widespread Eurasian species (*A. minutum, A. simplex*, and *A. strigosum*), similarly as resolved here for *A. szovitsianum* from Iran. Still, the existence of an independent Asian lineage cannot be ruled out either. Taxonomic identification of some Asian species, however, may be tentative, and thorough taxonomic revision of Asian species is necessarily needed as first. The complete Eurasian sampling in future studies may help to identify the missing parental species for some polyploids (see above), reinforce or weaken the phylogenetic signal in genome size, and will allow to gain deeper insights into the colonization and speciation processes in this group.

## Data Availability Statement

The datasets generated for this study can be found in online repositories. The names of the repository/repositories and accession number(s) can be found below: https://www.ncbi.nlm.nih.gov/genbank/, MW070213–MW070364; https://www.ncbi.nlm.nih.gov/genbank/, MW022541–MW022804; and https://www.ncbi.nlm.nih.gov/genbank/, MW070365–MW070517.

## Author Contributions

SŠ and JZ-L conceived and designed the study and wrote the manuscript. SŠ and VC sampled plant material. All authors generated data and performed data analyses. All authors have read, revised, and approved the final manuscript.

## Conflict of Interest

The authors declare that the research was conducted in the absence of any commercial or financial relationships that could be construed as a potential conflict of interest.
