# Supplementary material for: Multiple Drivers of High Species Diversity and Endemism Among Alyssum Annuals in the Mediterranean: The Evolutionary Significance of the Aegean Hotspot
Source: Front Plant Sci. 2021 Apr 27;12:627909. doi: 10.3389/fpls.2021.627909 (PMC8112278; doi:10.3389/fpls.2021.627909)
Supplement: Supplementary file 2 [file Table_2.PDF]

Supplementary Table S2. List of accessions and sequences (ITS of nrDNA) used for computation of the time-calibrated phylogenetic tree in BEAST. References: Koch et al. (1999) Plant Biology 1: 529-537; Warwick et al. (2008) Botany 86: 315–336; Warwick et al. (2010) Plant Systematics and Evolution 285: 209-232; German et al. (2009) Plant Systematics and Evolution 283: 33-56; Rešetnik et al. (2013) Molecular Phylogenetics and Evolution 69: 772-786; Salmerón-Sánchez et al. (2018) PLOS One 13: e0208307; Melichárková et al. (2019) American Journal of Botany 106: 1499-1518; Španiel et al. (2011) American Journal of Botany 98: 1887-1904; Španiel et al. (2017) Plant Systematics and Evolution 303: 1443-1465.

Accession or sequence identification codes used in the alignment and tree	Species name	Population or voucher codes (see the references in the column 'Source' for more details)	GenBank accession number	Source
ALB2, ALB3	Alyssum repens	281ALB	MK096792-MK096800	Melichárková et al. 2019
BAS18, BAS19	Alyssum montanum	95BAS	MK096801-MK096806	Melichárková et al. 2019
BEL1, BEL2, BEL3, BEL4	Alyssum repens	74BEL	MK096807-MK096810	Melichárková et al. 2019
BEL8, BEL9, BEL10, BEL11	Alyssum repens	574RUN	MK096811-MK096840	Melichárková et al. 2019
BOR6, BOR7	Alyssum bosniacum	199BOR	MK096841-MK096852	Melichárková et al. 2019
CAR1, CAR2	Alyssum repens	71CAR	MK096853-MK096863	Melichárková et al. 2019
CEA1, CEA2	Alyssum repens	282CEA	MK096864-MK096881	Melichárková et al. 2019
CRU2, CRU6	Alyssum repens	573CRU	MK096882-MK096883	Melichárková et al. 2019
FTE3	Alyssum minutum	160FTE	MK096884-MK096886	Melichárková et al. 2019
GRI1, GRI2	Alyssum repens	420GRI	MK096887-MK096890	Melichárková et al. 2019
HAG6	Alyssum alyssoides	43HAG	MK096891-MK096892	Melichárková et al. 2019
HOC1, HOC2	Alyssum gmelinii	421HOC	MK096893-MK096904	Melichárková et al. 2019
JAB1, JAB2	Alyssum vernale	42JAB	MK096905-MK096911	Melichárková et al. 2019
JHN1, JHN2	Alyssum repens	419JHN	MK096912-MK096914	Melichárková et al. 2019
KAR1, KAR2	Alyssum reiseri	40KAR	MK096915-MK096927	Melichárková et al. 2019
KIC6, KIC7	Alyssum moellendorfianum	120KIC	MK096928-MK096935	Melichárková et al. 2019
KIR1, KIR2	Alyssum repens	417KIR	MK096936-MK096939	Melichárková et al. 2019
KLA1, KLA2	Alyssum repens	132KLA	MK096940-MK096948	Melichárková et al. 2019
KLE1_rib1	Alyssum repens	415KLE	MK096949-MK096950	Melichárková et al. 2019
KRA1,KRA2	Alyssum gmelinii	52KRA	MK096951-MK096967	Melichárková et al. 2019
LAV1, LAV2	Alyssum repens	132KLA	MK096940-MK096948	Melichárková et al. 2019
LIP2, LIP3	Alyssum rostratum	22LIP	MK096979-MK096981	Melichárková et al. 2019
MAG7, MAG8	Alyssum spruneri	143MAG	MK096982-MK096992	Melichárková et al. 2019
MDN4, MDN13	Alyssum repens	571MDN	MK096993-MK097000	Melichárková et al. 2019
MUS3, MUS11	Alyssum repens	572MUS	MK097001-MK097003	Melichárková et al. 2019
OBE1, OBE2	Alyssum gmelinii	418OME	MK097004-MK097011	Melichárková et al. 2019
OBI1, OBI2	Alyssum wulfenianum subp. ovirense	423OBI	MK097012-MK097015	Melichárková et al. 2019
PEG1, PEG2	Alyssum repens	416PEG	MK097020-MK097025	Melichárková et al. 2019
PIA25, PIA26	Alyssum repens	73PIA	MK097026-MK097043	Melichárková et al. 2019
POS1, POS5	Alyssum repens	72POS	MK097044-MK097059	Melichárková et al. 2019
RET17, RET19	Alyssum repens	84RET	MK097060-MK097061	Melichárková et al. 2019
SAN10, SAN32	Alyssum gmelinii	94SAN	MK097062-MK097073	Melichárková et al. 2019

SKE1, SKE2	Alyssum vernale	27SKE	MK097074-MK097081	Melichárková et al. 2019
SUM1, SUM2	Alyssum vernale	143SUM	MK097082-MK097093	Melichárková et al. 2019
TOA1, TOA2	Alyssum repens	283TOA	MK097094-MK097105	Melichárková et al. 2019
POD6, POD7	Alyssum moellendorfianum	121POD	MK097106-MK097115	Melichárková et al. 2019
PRO6, PRO7	Alyssum bosniacum	200PRO	MK097116-MK097125	Melichárková et al. 2019
TOP6, TOP7	Alyssum spruneri	140TOP	MK097126-MK097133	Melichárková et al. 2019
diff58STE2	Alyssum diffusum subsp. diffusum	58STE	MW187015	this study (see also Španiel et al. 2011 for details of the population origin)
garg63MAR1	Alyssum diffusum subsp. garganicum	63MAR	MW187016	this study (see also Španiel et al. 2011 for details of the population origin)
hand169OLY2	Alyssum handelii	169OLY	MW187017	this study (see also Španiel et al. 2017 for details of the population origin)
pirin81VIH4p	Alyssum pirinicum	81VIHp	MW187018	this study (see also Španiel et al. 2017 for details of the population origin)
oro91TDE4	Alyssum orophilum	91TDE4	MW187019	this study (see also Španiel et al. 2011 for details of the population origin)
austrodalm101GRO1	Alyssum austrodalmaticum	101GRO	MW187020	this study (see also Španiel et al. 2017 for details of the population origin)
austrodalm119KOR9	Alyssum austrodalmaticum	119KOR	MW187021	this study (see also Španiel et al. 2017 for details of the population origin)
Alyssum_cacuminum	Alyssum cacuminum	T023 (France: Col de la Pierre-St.-Martin, Schonswetter & Tribsch 6467 (WU))	KF022530	Rešetnik et al. 2013 (under the name A. cuneifolium)
Alyssum_fastigiatum	Alyssum fastigiatum	T017 (Spain, Sierra Nevada, Morrón del Mediodía)	KF022573	Rešetnik et al. 2013 (under the name A. nevadense)
22LIP_al_4, 22LIP_al_5	Alyssum alyssoides	22LIP-al	MW022541-MW022542	this study
60AMA_al_1, 60AMA_al_2	Alyssum alyssoides	60AMA-al	MW022543-MW022544	this study
69IMP_al_2, 69IMP_al_3	Alyssum alyssoides	69IMP-al	MW022545-MW022548	this study
77DEV_mur_2, 77DEV_mur_3	Odontarrhena muralis	77DEV-mu	MW022549-MW022552	this study
90CEU_al_2, 90CEU_al_3	Alyssum alyssoides	90CEU-al	MW022553-MW022554	this study
109PRI_sx_3, 109PRI_sx_4	Alyssum simplex	109PRI-sx	MW022555-MW022556	this study
112SUT_sx_1, 112SUT_sx_3	Alyssum simplex	109PRI-sx	MW022557-MW022558	this study
114KRU_al_1, 114KRU_al_2	Alyssum alyssoides	114KRU-al	MW022559-MW022562	this study
131OPI_al_1, 131OPI_al_2	Alyssum alyssoides	114KRU-al	MW022563-MW022564	this study

151KAT_fo_1_9, 151KAT_fo_3_25	Alyssum foliosum	151KAT-fo	MW022565-MW022566	this study
151KAT_sx_6, 151KAT_sx_7	Alyssum simplex	151KAT-sx	MW022567-MW022572	this study
160FTE_sc_1, 160FTE_sc_8	Alyssum siculum	160FTE-sc	MW022573-MW022574	this study
163KROb_sc_4, 163KROb_sc_5	Alyssum siculum	163KROb-sc	MW022575-MW022579	this study
176VNS_st_3, 176VNS_st_6	Alyssum strigosum	176VNS-st	MW022580-MW022581	this study
244ORK_tt_1, 244ORK_tt_5	Odontarrhena tortuosa	244ORK-tt	MW022582-MW022591	this study
250BUR_al_1, 250BUR_al_2	Alyssum alyssoides	250BUR-al	MW022592-MW022595	this study
259SDS_al_1, 259SDS_al_3	Alyssum alyssoides	259SDS-al	MW022596-MW022601	this study
267ABR_gr_8, 267ABR_gr_10	Alyssum granatense	167ABR-gr	MW022602-MW022605	this study
279AKS_sx_1, 279AKS_sx_2	Alyssum simplex	279AKS-sx	MW022606-MW022607	this study
289TRJ_sz_2, 289TRJ_sz_3	Alyssum szovitsianum	289TRJ-sz	MW022608-MW022611	this study
304ENR_gr_1, 304ENR_gr_2	Alyssum granatense	304ENR-gr	MW022612-MW022617	this study
351GOR_co_1, 351GOR_co_2	Alyssum collinum	351GOR-co	MW022618-MW022623	this study
354OUK_gr_4, 354OUK_gr_10	Alyssum granatense	354OUK-gr	MW022624-MW022634	this study
360AZR_co_1, 360AZR_co_2	Alyssum collinum	360AZR-co	MW022635-MW022636	this study
367TZT_al_1, 367TZT_al_2	Alyssum alyssoides	367TZT-al	MW022637-MW022638	this study
368SFI_mi_1, 368SFI_mi_2	Alyssum minutum	368SFI-mi	MW022639-MW022640	this study
392DEM_hi_1_16, 392DEM_hi_2_16	Alyssum hirsutum	392DEM-hi	MW022641-MW022644	this study
410OGY_tu_5, 410OGY_tu_8	Alyssum turkestanicum	410OGY-tu	MW022645-MW022651	this study
424MET_sx_3, 424MET_sx_6	Alyssum simplex	424MET-sx	MW022652-MW022653	this study
429ORT_tu_1, 429ORT_tu_2	Alyssum turkestanicum	429ORT-tu	MW022654-MW022655	this study
443KAM_hi_1, 443KAM_hi_2	Alyssum hirsutum	443KAM-hi	MW022656-MW022658	this study
443KAM_mi_4, 443KAM_mi_7	Alyssum minutum	443KAM-mi	MW022659-MW022661	this study
449SRM_al_1, 449SRM_al_2	Alyssum alyssoides	449SRM-al	MW022662-MW022665	this study
457SVU_st_2, 457SVU_st_3	Alyssum strigosum	457SVU-st	MW022666-MW022669	this study
458PSS_mi_5, 458PSS_mi_6	Alyssum minutum	458PSS-mi	MW022670-MW022671	this study
458PSS_um_1, 458PSS_um_3	Alyssum umbellatum	458PSS-um	MW022672-MW022673	this study
469CHS_st_1, 469CHS_st_2	Alyssum strigosum	469CHS-st	MW022674-MW022675	this study
474RAE_st_1, 474RAE_st_2	Alyssum strigosum	474RAE-st	MW022676-MW022677	this study
481PTK_ss_1, 481PTK_ss_2	Alyssum simulans	481PTK-ss	MW022678-MW022684	this study
485ZIR_sc_1, 485ZIR_sc_3	Alyssum siculum	485ZIR-sc	MW022685-MW022689	this study
488OSK_sc_11, 488OSK_sc_15	Alyssum siculum	488OSK-sc	MW022690-MW022691	this study
489SAG_ss_1_25, 489SAG_ss_3_25	Alyssum simulans	489SAG-ss	MW022692-MW022693	this study
491GRN_fo_1_25, 491GRN_fo_2_9	Alyssum foliosum	491GRN-fo	MW022694-MW022697	this study
499THE_fo_1_9, 499THE_fo_1_16	Alyssum foliosum	499THE-fo	MW022698-MW022701	this study
499THE_sx_1, 499THE_sx_4	Alyssum simplex	499THE-sx	MW022702-MW022703	this study
507PZU_al_2, 507PZU_al_4	Alyssum alyssoides	507PZU-al	MW022704-MW022706	this study
507PZU_hi_9, 507PZU_hi_10	Alyssum hirsutum	507PZU-hi	MW022707-MW022708	this study
541IDA_mi_1A, 541IDA_mi_3B	Alyssum minutum	541IDA-mi	MW022709-MW022710	this study
541IDA_sc_1, 541IDA_sc_2	Alyssum siculum	541IDA-sc	MW022711-MW022712	this study
543ARN_sx_1, 543ARN_sx_2	Alyssum simplex	543ARN-sx	MW022713-MW022714	this study

544DTI_mi_3, 544DTI_mi_4	Alyssum minutum	544DTI-mi	MW022715-MW022718	this study
544DTI_sc_9, 544DTI_sc_10	Alyssum siculum	544DTI-sc	MW022719-MW022720	this study
544DTI_ss_3, 544DTI_ss_4	Alyssum simulans	544DTI-ss	MW022721-MW022725	this study
544DTI_sy_2, 544DTI_sy_3	Alyssum smyrnaeum	544DTI-sy	MW022726-MW022727	this study
546LZR_fo_6, 546LZR_fo_8	Alyssum foliosum	546LZR-fo	MW022728-MW022731	this study
546LZR_ss_1B, 546LZR_ss_1C	Alyssum simulans	546LZR-ss	MW022732-MW022737	this study
546LZR_sy_2A, 546LZR_sy_2B	Alyssum smyrnaeum	546LZR-sy	MW022738-MW022741	this study
547DCH_sc_4, 547DCH_sc_9	Alyssum siculum	547DCH-sc	MW022742-MW022743	this study
549QUA_sc_1, 549QUA_sc_2	Alyssum siculum	549QUA-sc	MW022744-MW022746	this study
550BTG_sc_1, 550BTG_sc_2	Alyssum siculum	549QUA-sc	MW022747-MW022750	this study
551RBS_sx_1, 551RBS_sx_2	Alyssum simplex	551RBS-sx	MW022751-MW022752	this study
553HRH_al_9, 553HRH_al_10	Alyssum alyssoides	553HRH-al	MW022753-MW022756	this study
579MER_gr_1, 579MER_gr_2	Alyssum granatense	579MER-gr	MW022757-MW022763	this study
580ETR_co_1, 580ETR_co_2	Alyssum collinum	580ETR-co	MW022764-MW022766	this study
583AGG_pg_1, 583AGG_pg_2	Alyssum pogonocarpum	583AGG-pg	MW022767-MW022768	this study
584ATA_fo_1, 584ATA_fo_3	Alyssum foliosum	584ATA-fo	MW022769-MW022770	this study
585SFI_sx_1, 585SFI_sx_2	Alyssum simplex	585SFI-sx	MW022771-MW022772	this study
588SNT_um_1, 588SNT_um_2	Alyssum umbellatum	588SNT-um	MW022773-MW022774	this study
590SKL_um_1, 590SKL_um_2	Alyssum umbellatum	590SKL-um	MW022775-MW022776	this study
592PTR_fo_1, 592PTR_fo_2	Alyssum foliosum	592PTR-fo	MW022777-MW022779	this study
592PTR_mi_1, 592PTR_mi_2	Alyssum minutum	592PTR-mi	MW022780-MW022782	this study
592PTR_xi_1, 592PTR_xi_2	Alyssum xiphocarpum	592PTR-xi	MW022783-MW022786	this study
594PEO_fu_9, 594PEO_fu_10	Alyssum fulvescens	594PEO-fu	MW022787-MW022792	this study
595KEK_fu_5, 595KEK_fu_6	Alyssum fulvescens	595KEK-fu	MW022793-MW022800	this study
595KEK_sx_1, 595KEK_sx_2	Alyssum simplex	595KEK-sx	MW022801-MW022802	this study
596AML_sy_1, 596AML_sy_2	Alyssum smyrnaeum	596AML-sy	MW022803-MW022804	this study
Odontarrhena_corymbosoidea	Odontarrhena corymbosoidea	Micevski, ZA	KF022529	Rešetnik et al. 2013
Odontarrhena_borzaeana	Odontarrhenaborzaeana	Rešetnik, ZA	KF022525	Rešetnik et al. 2013
Odontarrhena_chalcidica	Odontarrhena chalcidica	Rešetnik, ZA	KF022526	Rešetnik et al. 2013
Odontarrhena_condensata_flexibilis	Odontarrhena condensata subsp. flexibilis	Eren & Parolly 7550, B 100208573	KF022527	Rešetnik et al. 2013
Odontarrhena_gevgelicensis	Odontarrhena gevgelicensis	Micevski, MKNH	KF022538	Rešetnik et al. 2013
Odontarrhena_kavadarcensis	Odontarrhena kavadarcensis	Micevski, MKNH	KF022544	Rešetnik et al. 2013
Odontarrhena_serpentina	Odontarrhena serpentina	Micevski, MKNH	KF022582	Rešetnik et al. 2013
Odontarrhena alpestris	Odontarrhena alpestris	Gutermann (WU)	KF022515	Rešetnik et al. 2013
Odontarrhena_serpyllifolia	Odontarrhena serpyllifolia	Plazibat, ZA	KF022583	Rešetnik et al. 2013
Odontarrhena_skopjensis	Odontarrhena skopjensis	Micevski, ZA	KF022594	Rešetnik et al. 2013
Odontarrhena_markgrafii	Odontarrhena markgrafii	Rešetnik, ZA	KF022548	Rešetnik et al. 2013
Odontarrhena_muralis	Odontarrhena muralis	Frajman, Schönswetter & Bardy, ZA	KF022568	Rešetnik et al. 2013
Odontarrhena_nebrodensis	Odontarrhena nebrodensis	Brullo, CAT 041739	KF022572	Rešetnik et al. 2013
Odontarrhena_tortuosa	Odontarrhena tortuosa	Cigić & Boršić, ZA	KF022604	Rešetnik et al. 2013

Meniocus_aureus	Meniocus aureus	Misirdali, Orcan	KF022523	Rešetnik et al. 2013
Meniocus_linifolius	Meniocus linifolius	Schönswetter & Tribsch 6607, WU	KF022546	Rešetnik et al. 2013
Meniocus_meniocoides	Meniocus meniocoides	Samuelsson, s.n., 05 July 1933 (MO)	EF514612	Warwick et al. 2008
Alyssum_aurantiacum	Alyssum aurantiacum	Döring, Parolly & Tolimir 697b, B100132695	KF022522	Rešetnik et al. 2013
Alyssum_baumgartnerianum	Alyssum baumgartnerianum	Nazeri, All, ZA	KF022524	Rešetnik et al. 2013
Alyssum_corningii	Alyssum corningii	Faghihnia, Zangooei	KF022528	Rešetnik et al. 2013
Alyssum_dasycarpum	Alyssum dasycarpum	Orcan, Ocak	KF022531	Rešetnik et al. 2013
Alyssum_doerfleri	Alyssum doerfleri	Micevski, ZA	KF022537	Rešetnik et al. 2013
Alyssum_harputicum	Alyssum harputicum	Joharchi, ZA	KF022540	Rešetnik et al. 2013
Alyssum_lenense	Alyssum lenense	Byalt, s.n., 24 May 1993 (MO)	EF514610	Warwick et al. 2008
Alyssum_lepidotostellatum	Alyssum lepidotostellatum	Nazari, ZA	KF022545	Rešetnik et al. 2013
Alyssum_misirdalianum	Alyssum misirdalianum	Binzet, ANK	KF022552	Rešetnik et al. 2013
Alyssum_niveum	Alyssum niveum	Ekim, Aytac, Dunan	KF022574	Rešetnik et al. 2013
Alyssum_paphlagonicum	Alyssum paphlagonicum	Joharchi, ZA	KF022577	Rešetnik et al. 2013
Alyssum_persicum	Alyssum persicum	Schönswetter & Tribsch 6984, WU	KF022578	Rešetnik et al. 2013
Alyssum_sulphureum	Alyssum sulphureum	Nazari (ZA)	KF022524	Rešetnik et al. 2013
Alyssum_tetrastemon	Alyssum tetrastemon	Nazari (ZA)	KF022602	Rešetnik et al. 2013
Alyssum_thymops	Alyssum thymops	Nazari (ZA)	KF022603	Rešetnik et al. 2013
Aurinia_corymbosa	Aurinia corymbosa	Niketić, Stevanović, Tomović, Vukojičić, BEO	KF022607	Rešetnik et al. 2013
Aurinia_gionae	Aurinia gionae	Gutermann 35748, herb. Gutermann	KF022609	Rešetnik et al. 2013
Aurinia_leucadea_diomedea	Aurinia leucadea subsp. diomedea	Bogdanović, ZA	KF022610	Rešetnik et al. 2013
Aurinia_moreana	Aurinia moreana	Horandl & Hadaček jun. & sen., W-1998–4073	KF022616	Rešetnik et al. 2013
Aurinia_petraea	Aurinia petraea	Dakskobler, ZA	KF022617	Rešetnik et al. 2013
Aurinia_saxatilis	Aurinia saxatilis	Alegro, ZA	KF022621	Rešetnik et al. 2013
Aurinia_sinuata	Aurinia sinuata	Plazibat, ZA	KF022627	Rešetnik et al. 2013
Berteroa_incana	Berteroa incana	Schönswetter & Tribsch 6285, WU	KF022630	Rešetnik et al. 2013
Berteroa_mutabilis	Berteroa mutabilis	Alegro & Bogdanović, ZA	KF022631	Rešetnik et al. 2013
Berteroa_obliqua	Berteroa obliqua	Gutermann 35727, herb. Gutermann	KF022633	Rešetnik et al. 2013
Berteroa_orbiculata	Berteroa orbiculata	Miladinova, MKNH	KF022634	Rešetnik et al. 2013
Bornmuellera_baldaccii	Bornmuellera baldaccii	Eisenblätter & Willing 84137, B 100341181	KF022635	Rešetnik et al. 2013
Bornmuellera_cappadocica	Bornmuellera cappadocica	Sorger, W- 1992–8212	KF022636	Rešetnik et al. 2013
Bornmuellera_dieckii	Bornmuellera dieckii	Hundosi, MKNH	KF022637	Rešetnik et al. 2013
Bornmuellera_tymphaea	Bornmuellera tymphaea	Gutermann 35075, WU	KF022639	Rešetnik et al. 2013
BornmuelleraxLeptoplax	Bornmuellera x Leptoplax	Gutermann 35072, herb. Gutermann	KF022641	Rešetnik et al. 2013
Bornmuellera_emarginata	Bornmuellera emarginata	Gutermann 35073, herb. Gutermann	KF022666	Rešetnik et al. 2013
Bornmuellera_davisii	Bornmuellera davisii	A. Tali; (E 5806)	GQ497875	Warwick et al. 2010
Degenia_velebitica	Degenia velebitica	Liber, ZA	KF022646	Rešetnik et al. 2013

Acuston_lunarioides	Acuston lunarioides	Burri & Krendl, W-2000-11251	KF022652	Rešetnik et al. 2013
Brachypus_suffruticosus	Brachypus suffruticosus	Mummenhoff	FM164657	German et al. 2009
Resetnikia_triquetra	Resetnikia triquetra	Rešetnik, ZA	KF022655	Rešetnik et al. 2013
Irania_umbellata	Irania umbellata	Khosravi, Biglari	KF022656	Rešetnik et al. 2013
Hormathophylla_cadevalliana	Hormathophylla cadevalliana	Mahimón	KM033814	Salmerón-Sánchez et al. 2018
Hormathophylla_cochleata	Hormathophylla cochleata subsp. cochleata	Agoudal	KM033792	Salmerón-Sánchez et al. 2018
Hormathophylla_ligustica	Hormathophylla ligustica	Trinità di Entracque	KM033759	Salmerón-Sánchez et al. 2018
Hormathophylla_lapeyrouseana	Hormathophylla lapeyrouseana	Monteagudo de las Vicarias	KM033741	Salmerón-Sánchez et al. 2018
Hormathophylla_longicaulis	Hormathophylla longicaulis	Boca de la Pesca	KM033820	Salmerón-Sánchez et al. 2018
Hormathophylla_saxigena	Hormathophylla saxigena	Quillan	KM033757	Salmerón-Sánchez et al. 2018
Hormathophylla_pyrenaica	Hormathophylla pyrenaica	Nohèdes	KM033761	Salmerón-Sánchez et al. 2018
Hormathophylla_reverchonii	Hormathophylla reverchonii	Sierra de la Cabrilla	KM033821	Salmerón-Sánchez et al. 2018
Hormathophylla_spinosa	Hormathophylla spinosa	Prados del Rey	KM033777	Salmerón-Sánchez et al. 2018
Hormathophylla_purpurea	Hormathophylla purpurea	Los Cauchiles	KM033733	Salmerón-Sánchez et al. 2018
Phyllolepidum_cyclocarpum	Phyllolepidum cyclocarpum	Döring, Parolly & Tolimir 1110, B100132672	KF022667	Rešetnik et al. 2013
Phyllolepidum_rupestre	Phyllolepidum rupestre	Gutermann 35182, WU	KF022669	Rešetnik et al. 2013
Physoptychis_caspica	Physoptychis caspica	Khosravi (ZA)	KF022671	Rešetnik et al. 2013
Clastopus_vestitus	Clastopus vestitus	K. H. Rechinger & F. Rechinger 6461, B100209496	KF022642	Rešetnik et al. 2013
Galitzkya_macrocarpa	Galitzkya macrocarpa	Wesche, HAL	KF022657	Rešetnik et al. 2013
Galitzkya_potaninii	Galitzkya potaninii	Wesche, HAL	KF022659	Rešetnik et al. 2013
Galitzkya_spathulata	Galitzkya spathulata	Karelin, s.n., 1842 (MO)	EF514657	Warwick et al. 2008
Lepidotrichum_uechtritzianum	Lepidotrichum uechtritzianum	Stohr s.n., B 10 0209493	KF022665	Rešetnik et al. 2013
Clypeola_aspera	Clypeola aspera	Schönswetter & Tribsch 3861 (WU)	KF022643	Rešetnik et al. 2013
Clypeola_cyclodontea	Clypeola cyclodontea	Dubuis 14072 (MO)	EF514643	Warwick et al. 2008
Clypeola_dichotoma	Clypeola dichotoma	Strashafr & Reed, W- 21437	KF022644	Rešetnik et al. 2013
Clypeola_jonthlaspi	Clypeola jonthlaspi	Schönswetter & Tribsch 3860, WU	KF022645	Rešetnik et al. 2013
Clypeola_lappacea	Clypeola lappacea	Grant 15538 (MO)	EF514645	Warwick et al. 2008
Fibigia_clypeata	Fibigia clypeata	Bogdanović, ZA	KF022650	Rešetnik et al. 2013
Fibigia_macrocarpa	Fibigia macrocarpa	Döring, Parolly & Tolimir 1315, B100132627	KF022654	Rešetnik et al. 2013
Fibigia_clypeata_eriocarpa	Fibigia clypeata subsp. eriocarpa	Hein 45–5, B 10 0209827	KF022651	Rešetnik et al. 2013
Alyssoides_utriculata	Alyssoides utriculata	Gutermann 35181, HAL	KF022514	Rešetnik et al. 2013
Lutzia_cretica	Lutzia cretica	Strudle and Pokorny, s.n., 16 Apr. 1982 (MO)	EF514592	Warwick et al. 2008
Cuprella_antiatlantica	Cuprella antiatlantica	AANT01	KM033824	Salmerón-Sánchez et al. 2018
Cuprella_homalocarpa	Cuprella homalocarpa	AHOM01	KR269770	Salmerón-Sánchez et al. 2018
OUT_Arabidopsis_thaliana_AJ232900	Arabidopsis thaliana	ABRC, Columbia ecotype, Ohio State University	AJ232900	Koch et al. 1999

OUT_Clausia_aprica_DQ357529	Clausia aprica	Schischkin 1-June, 1931 (LE)	DQ357529	Warwick et al. 2008
