# Supplementary material for: Multiple Drivers of High Species Diversity and Endemism Among Alyssum Annuals in the Mediterranean: The Evolutionary Significance of the Aegean Hotspot
Source: Front Plant Sci. 2021 Apr 27;12:627909. doi: 10.3389/fpls.2021.627909 (PMC8112278; doi:10.3389/fpls.2021.627909)

Supplementary Figure 1. Phylogenetic reconstructions based on the *rpoB-trnC* sequences of cpDNA for the studied *Alyssum* species. **A.** Maximum-likelihood tree. Values above the branches are bootstrap support (BS) ≥50%; those below the branches are Bayesian posterior probability (BPP) values ≥0.75. The terminal sequence labels include the population code following **Supplementary Table 1** and an individual number. Branches and clades are coloured according to the species assignment. The species names next to the clades are followed by ploidy level and geographic occurrence in brackets (the country abbreviations follow **Figure 1**). The geographic origin of the two populations of diploid *A. fulvescens* exhibiting genetic divergence in each of the three DNA regions is also indicated (Samos and Chios islands). Numbers 1-6 in boxes indicate the main clades referred to in the text.

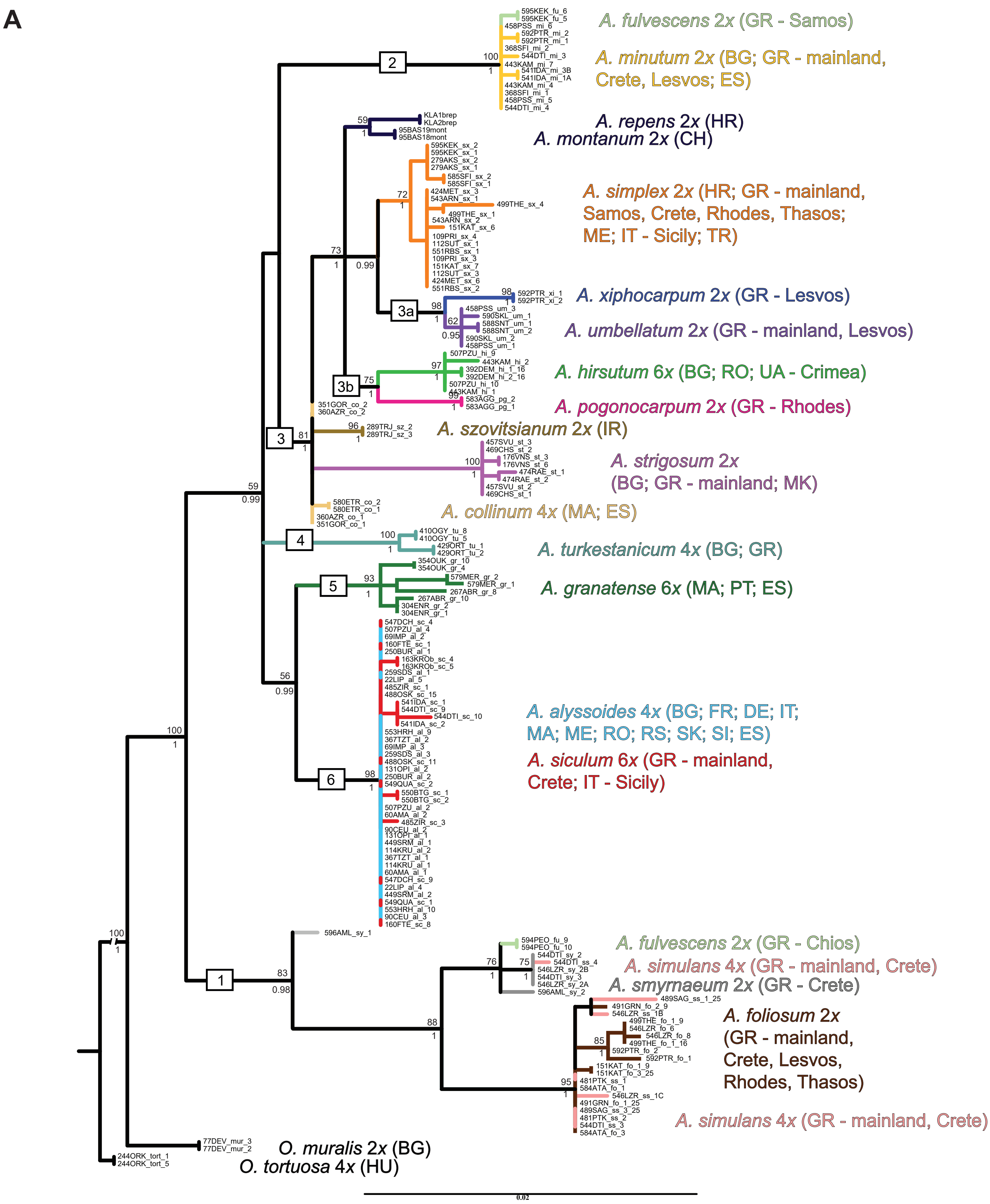

Supplementary Figure 1. Phylogenetic reconstructions based on the *rpoB-trnC* sequences of cpDNA for the studied *Alyssum* species.
B. NeighborNet diagram with branches coloured according to the species assignment, omitting the terminal labels of individual sequences for the sake of readability.
The species name is followed by its ploidy level in brackets. The geographic origin of the two populations of diploid *A. fulvescens* exhibiting genetic divergence in each of the three DNA regions is indicated (Samos and Chios islands). Numbers 1-6 in boxes indicate splits, which coincide with the supported clades resolved on the ML tree.

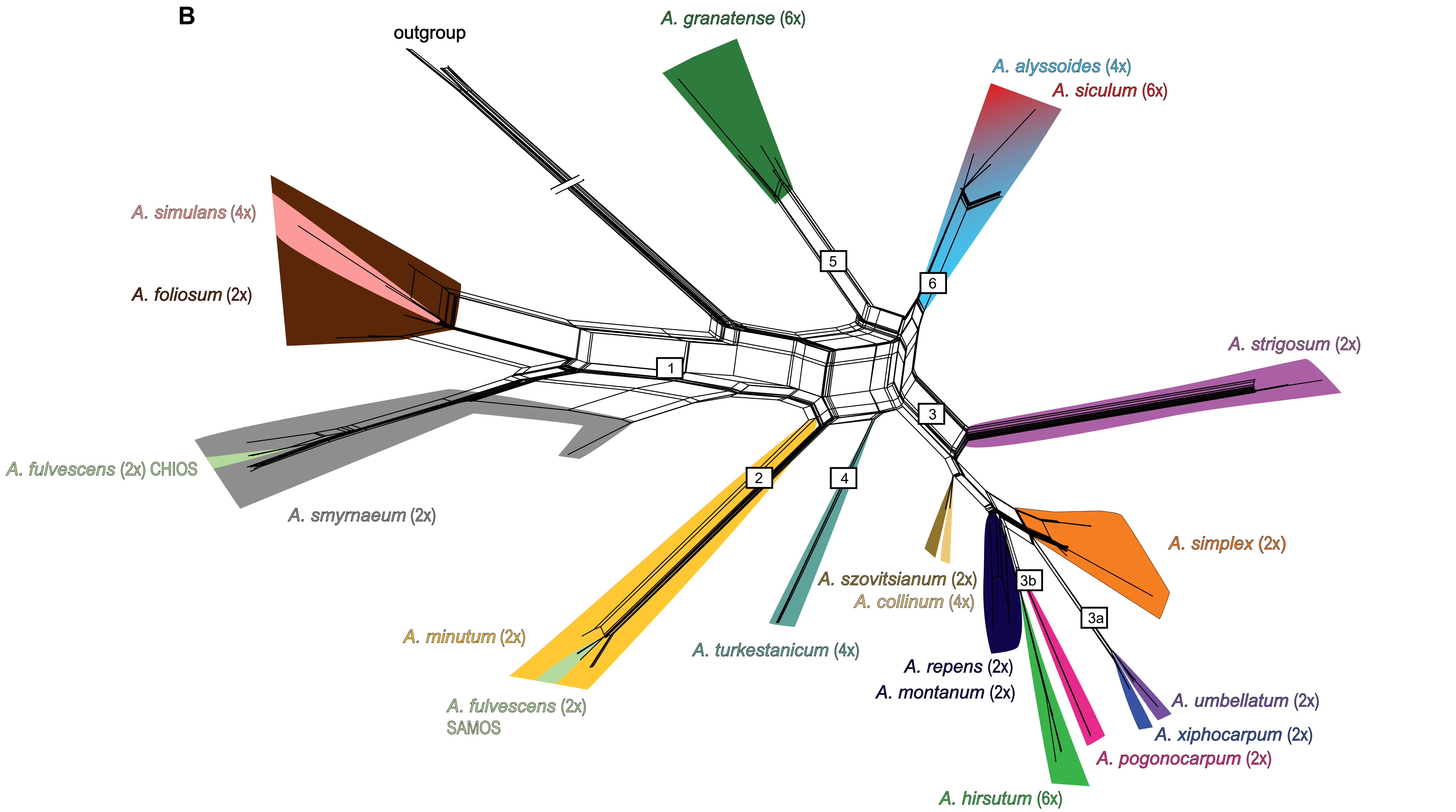

Supplementary Figure 2. Phylogenetic reconstructions based on the ITS sequences of nrDNA for the studied *Alyssum* species. **A.** Maximum-likelihood tree. Divergent ITS sequences observed in the tetraploid *A. simulans*, placed in distinct clades (nr. 3 and 4), are marked here as *A. simulans* 1 and *A. simulans* 2. Numbers 1-5 in boxes indicate the main clades referred to in the text. For more detailed tree description, see the legend of **Supplementary Figure 1A**.

A

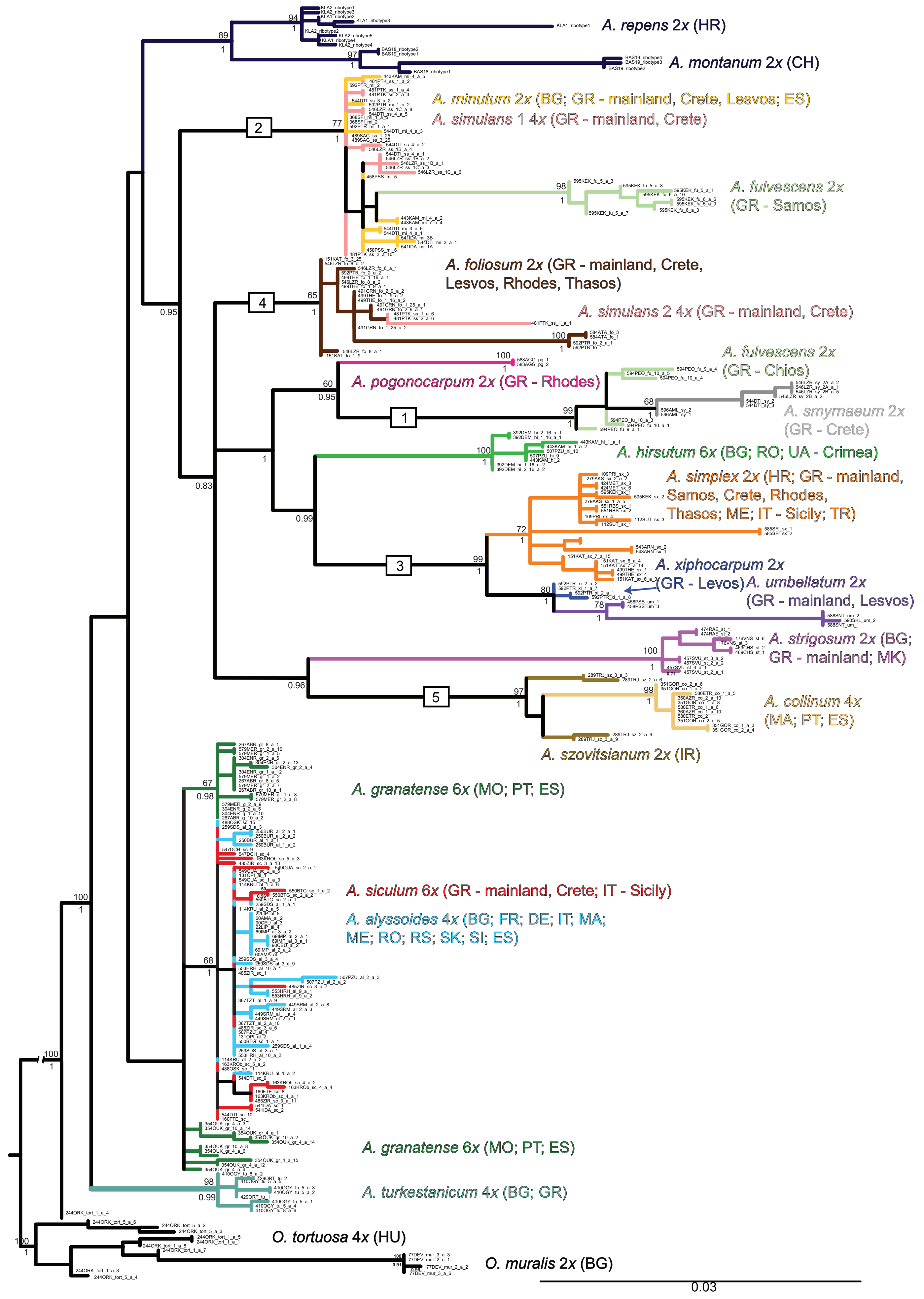

Supplementary Figure 2. Phylogenetic reconstructions based on the ITS sequences of nrDNA for the studied *Alyssum* species.
B. NeighborNet diagram with branches coloured according to the species assignment, omitting the terminal labels of individual sequences for the sake of readability. The species name is followed by its ploidy level in brackets. The geographic origin of the two populations of diploid *A. fulvescens* exhibiting genetic divergence in each of the three DNA regions is indicated (Samos and Chios islands). Numbers 1-5 in boxes indicate splits, which coincide with the supported clades resolved on the ML tree.

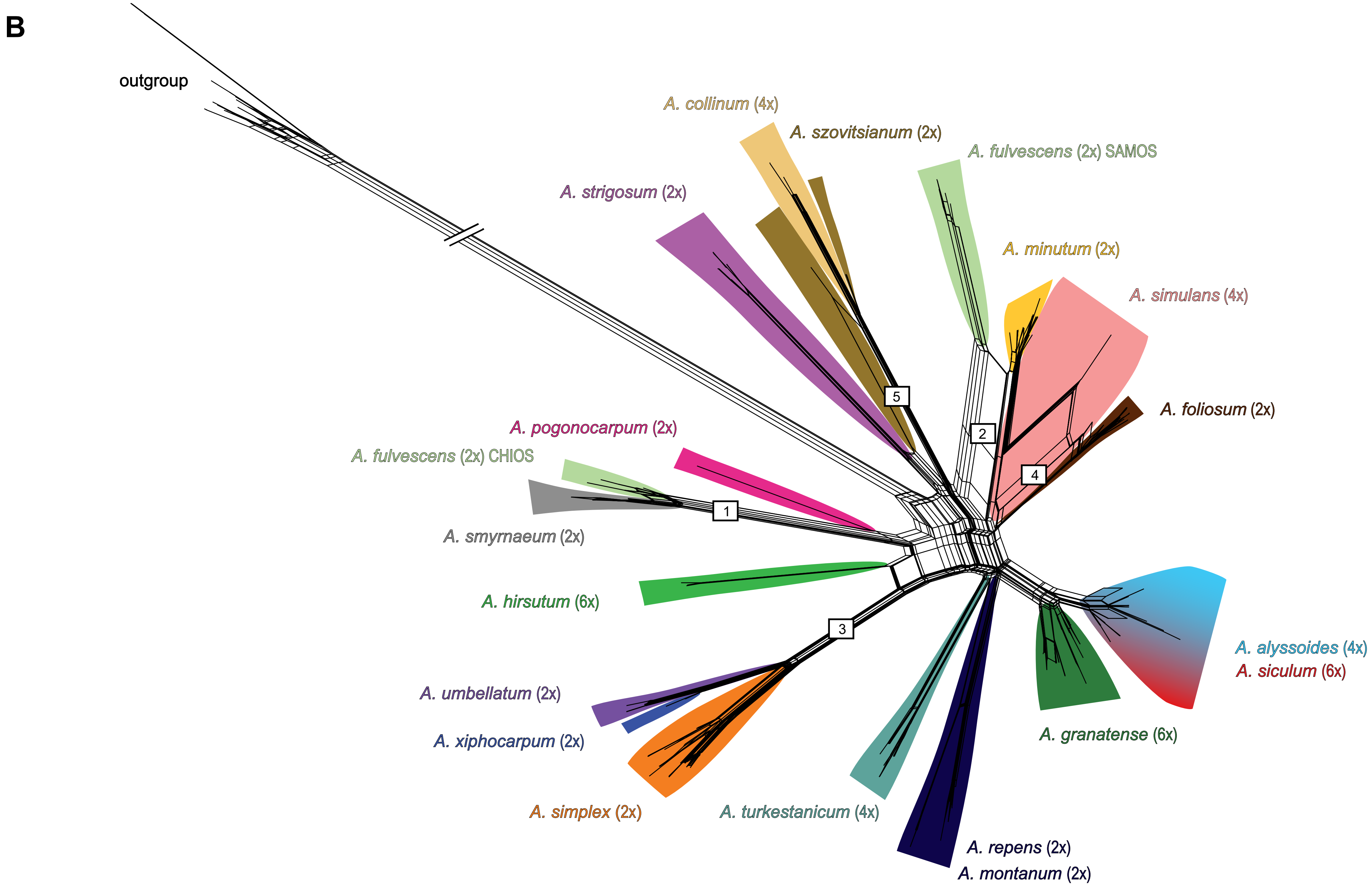

Supplementary Figure 3. Phylogenetic reconstructions based on the *DET1* sequence data of the studied *Alyssum* species. **A**. Maximum-likelihood tree. For more detailed tree description, see the legend of **Supplementary Figure 1A**. The numbers 1, 2 and 3 indicated next to the polyploid species names stand for different homeologs. In addition, symbols (pentagons and diamonds) highlight the alleles that deviated from the observed homeolog variation in *A. turkestanicum* and *A. hirsutum*, respectively. Numbers 1-9 in boxes indicate the main clades referred to in the text.

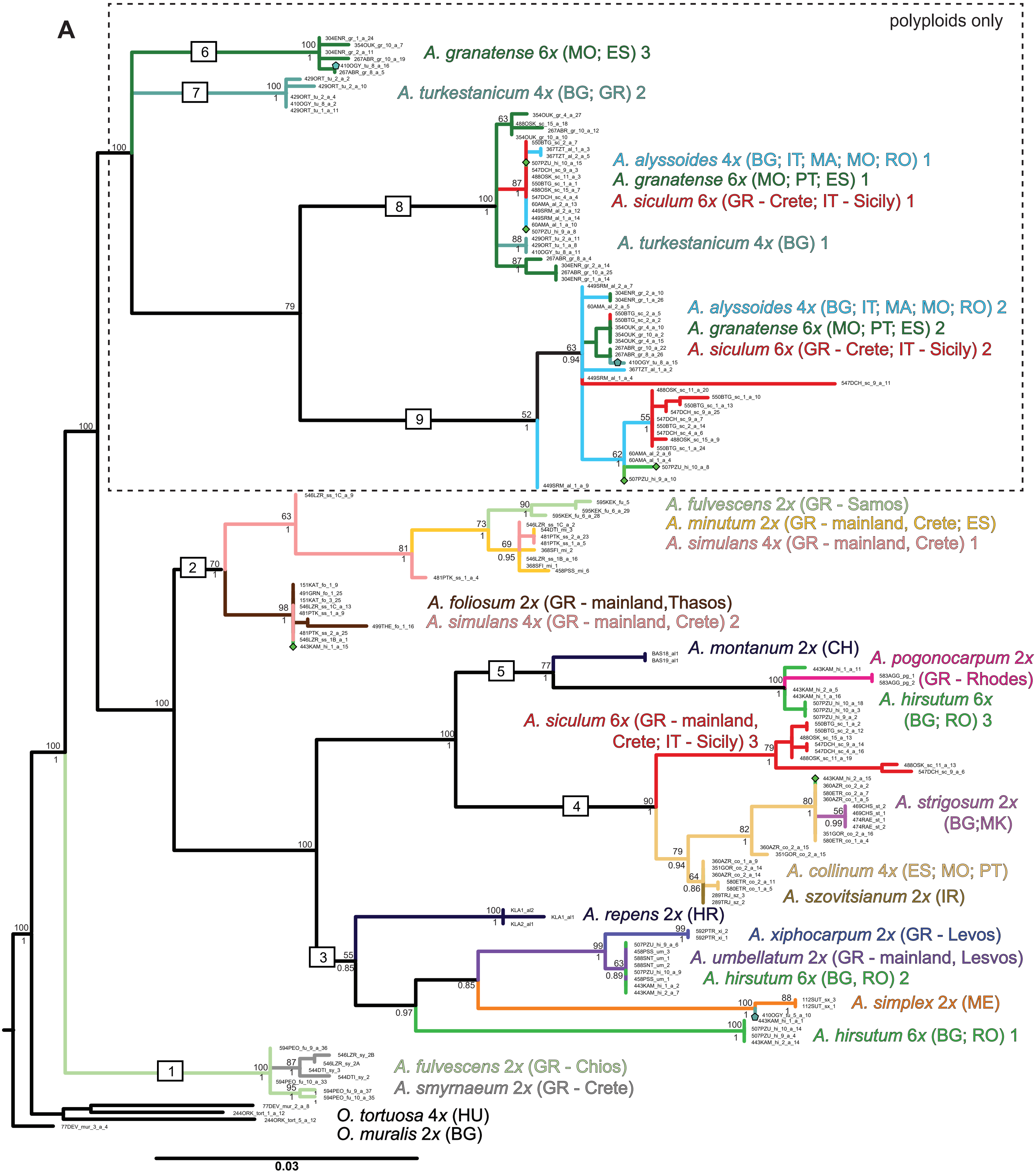

Supplementary Figure 3. Phylogenetic reconstructions based on the *DET1* sequence data of the studied *Alyssum* species. **B**. NeighborNet diagram with branches coloured according to the species assignment, omitting the terminal labels of individual sequences for the sake of readability. The species name is followed by its ploidy level. Diploid species names are underlined. The numbers 1, 2 and 3 in bold, indicated next to the polyploid species names, stand for different homeologs. In addition, symbols (pentagons and diamonds) highlight the alleles that deviated from the observed homeolog variation in *A. turkestanicum* and *A. hirsutum*, respectively. The geographic origin of the two populations of diploid *A. fulvescens* exhibiting genetic divergence in each of the three DNA regions is indicated (Samos and Chios islands). Numbers 1-9 in boxes indicate the main splits, which coincide well with the clades resolved on the ML tree.

B

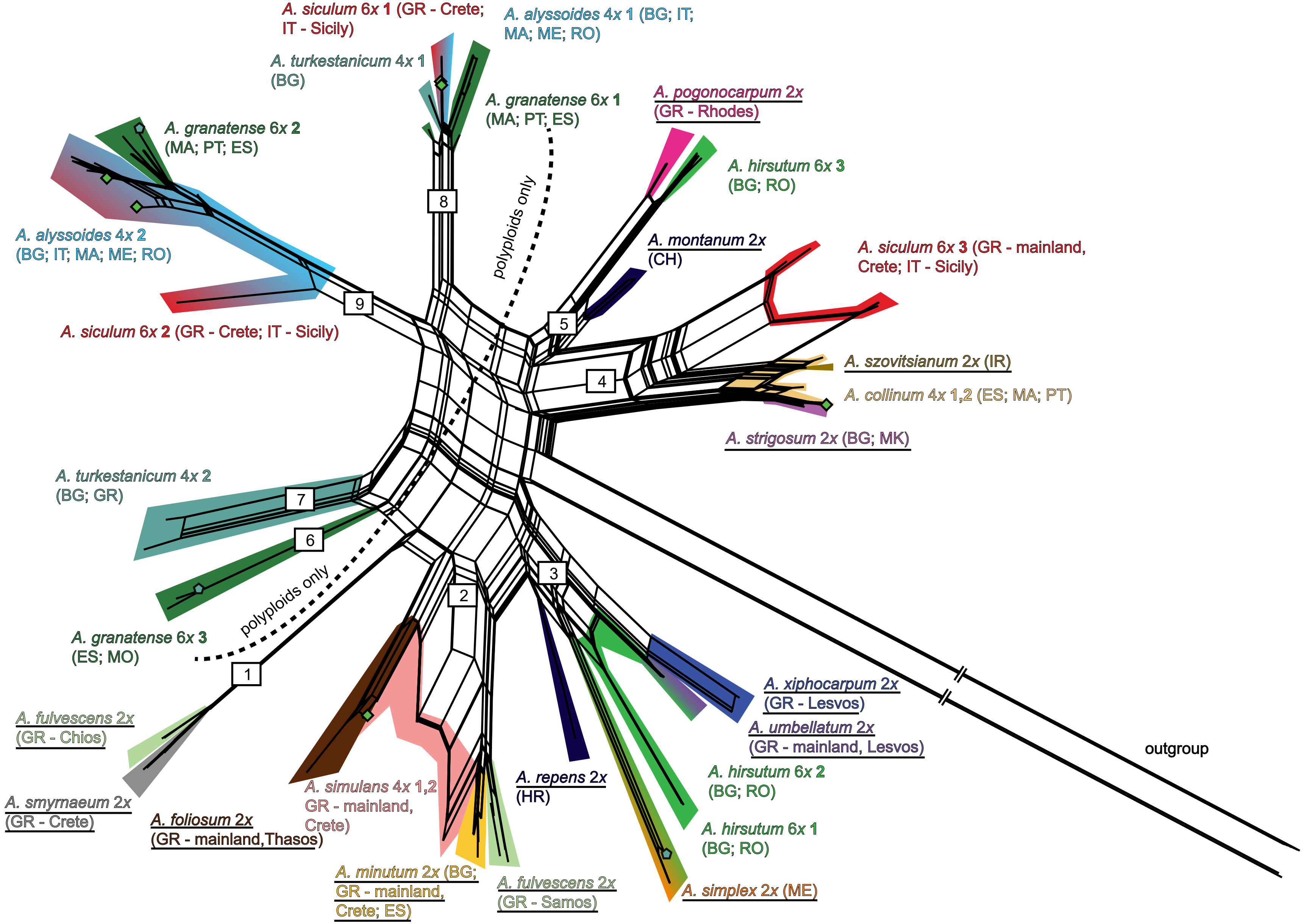

Supplementary Figure 4. Maximum clade credibility tree generated from the relaxed molecular-clock analysis in BEAST based on ITS sequence data of the tribe Alysseae, plus two outgroup species *(Arabidopsis thaliana*, tribe Camelineae; *Clausia aprica*, tribe Dontostemonea) used for secondary calibration points, denoted as B and C, following Huang et al. (2020), Annals of Botany 125: 29-47.
The simplified tree with the collapsed monophyletic clades at the level of *Alysseae* genera or *Alyssum* species is depicted on the first page, followed by the complete tree of all accessions on the second page.
The clades of the genus *Alyssum* are zoomed in on the next pages, labelled as A-D. The here studied annuals are shown on pages A, B and D; the nested *A. montanum-A. repens* perennial species complex is depicted on C and in more details on C1-C3. Time scale is given in millions of years. Horizontal blue bars represent 95% HPD intervals of divergence times; node values are median of divergence time estimates. Values below branches show Bayesian posterior probability (BPP) values ≥0.75 (omitted for the most terminal, intraspecific clades). See **Supplementary Table 2** for the list of accessions and sequences used.

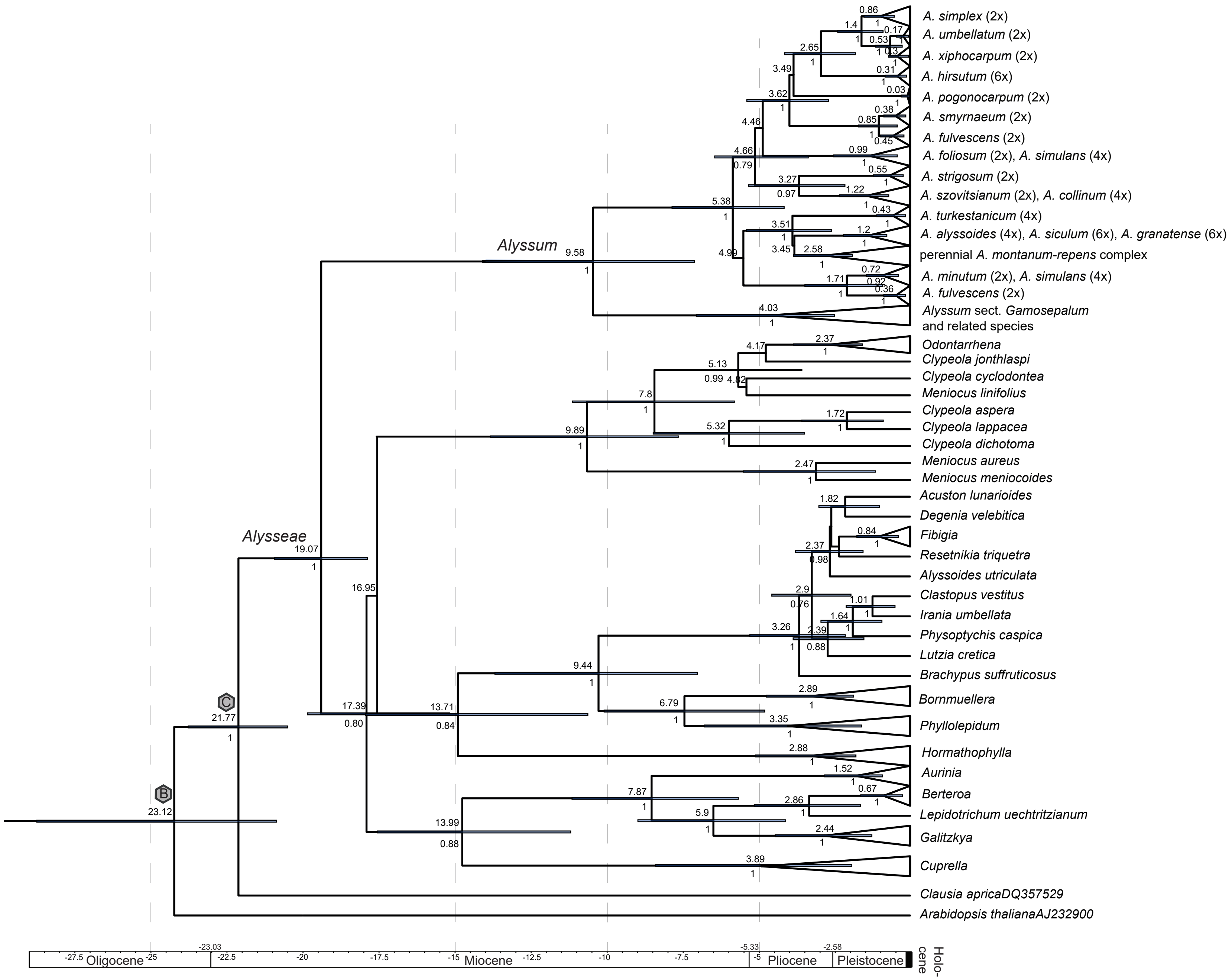

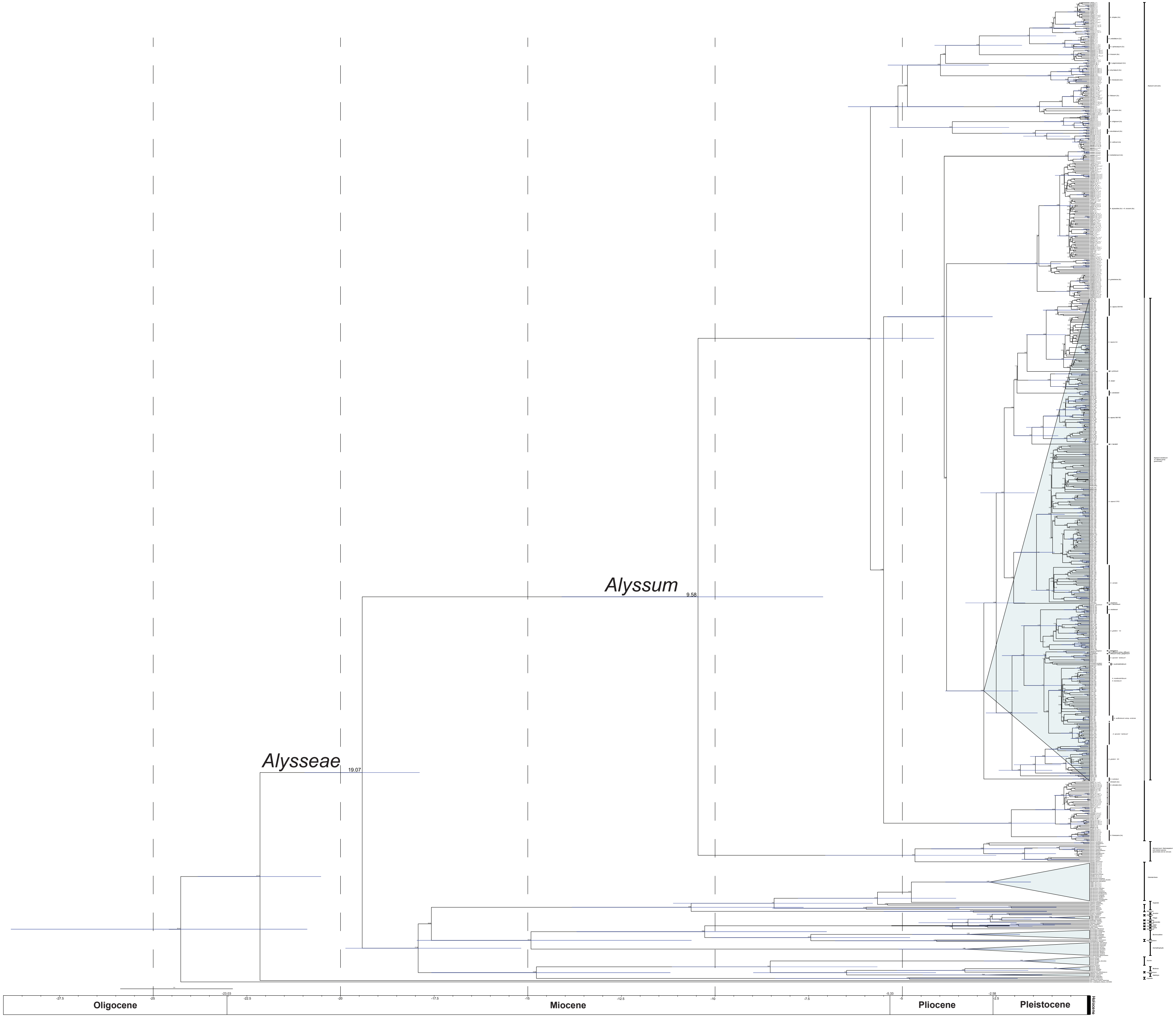
Alysseae
19.07
Alyssum
9.58
-27.5
-25
-23.03
-22.5
-20
-17.5
-15
-12.5
-10
-7.5
-5.33
-5
-2.58
-2.5
Oligocene
Miocene
Pliocene
Pleistocene
Holocene

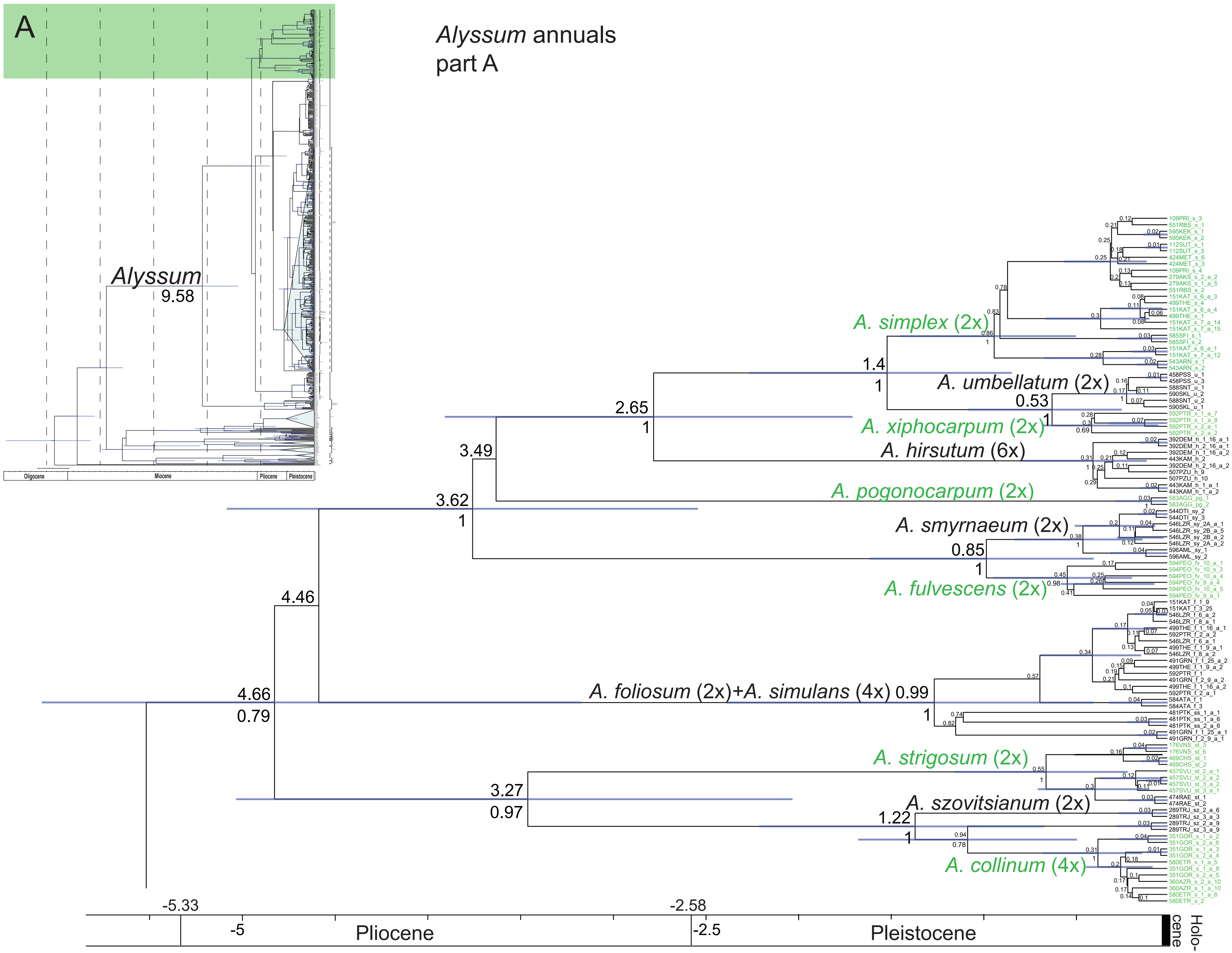

A
Alyssum
9.58
Oligocene
Miocene
Pliocene
Pleistocene
Alyssum annuals
part A
A. simplex (2x)
1.4
1
A. umbellatum (2x)
0.53
1
A. xiphocarpum (2x)
2.65
1
A. hirsutum (6x)
3.49
A. pogonocarpum (2x)
3.62
1
A. smyrnaeum (2x)
0.85
1
A. fulvescens (2x)
4.46
4.66
0.79
A. foliosum (2x)+A. simulans (4x) 0.99
1
A. strigosum (2x)
3.27
0.97
A. szovitsianum (2x)
1.22
1
A. collinum (4x)
-5.33
-2.58
-5
-2.5
Pliocene
Pleistocene
Holo-
cene

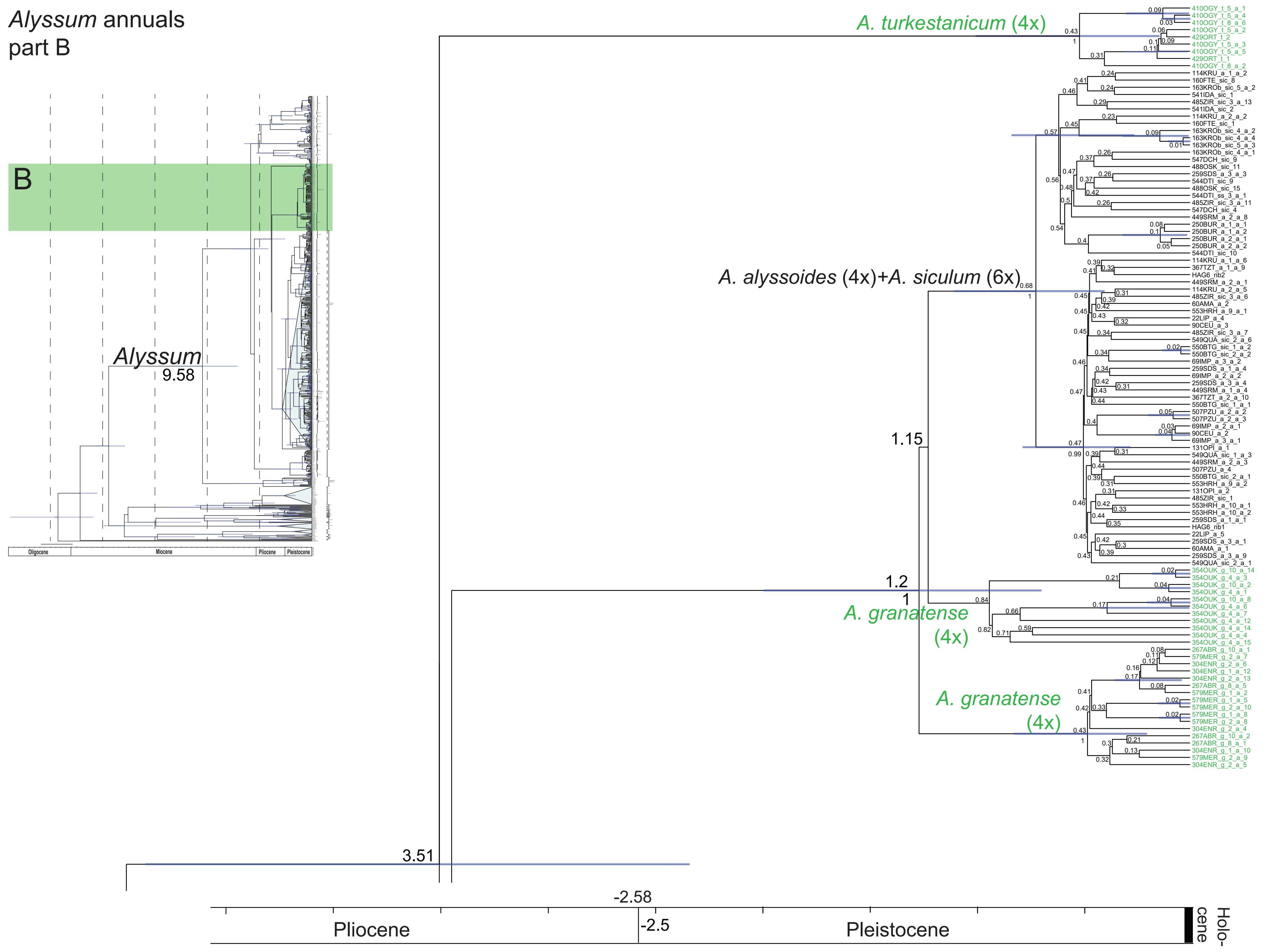
Alyssum annuals
part B
B
Alyssum
9.58
Oligocene
Miocene
Pliocene
Pleistocene
A. turkestanicum (4x)
A. alyssoides (4x)+A. siculum (6x)
1.15
1.2
1
A. granatense
(4x)
A. granatense
(4x)
3.51
-2.58
-2.5
Pliocene
Pleistocene
Holo-
cene

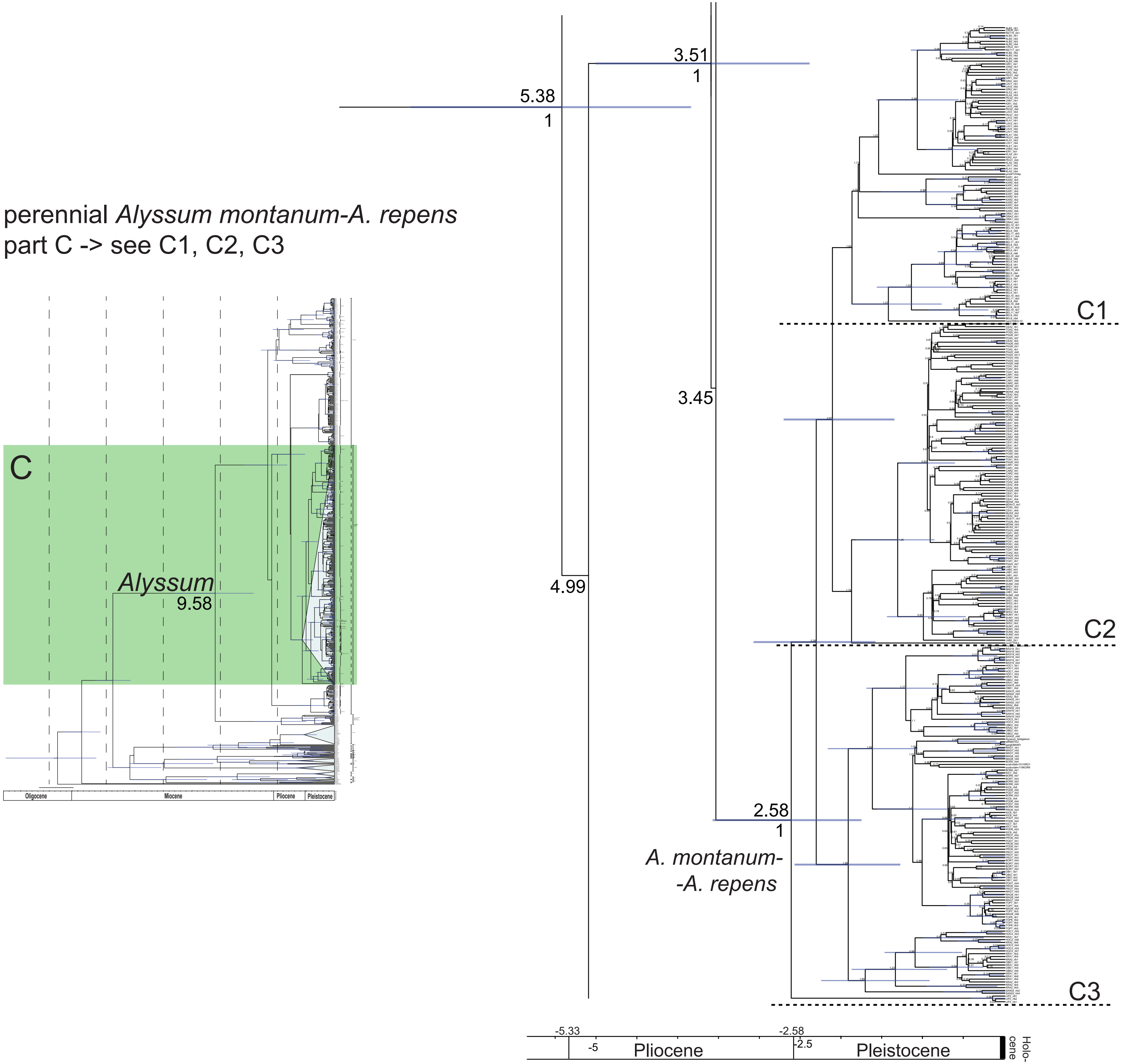
perennial *Alyssum montanum-A. repens*
part C -> see C1, C2, C3
C
Alyssum
9.58
Oligocene
Miocene
Pliocene
Pleistocene
5.38
1
3.51
1
4.99
3.45
2.58
1
A. montanum-
-A. repens
C1
C2
C3
-5.33
-5
-2.58
-2.5
Pliocene
Pleistocene
Holo-
cene

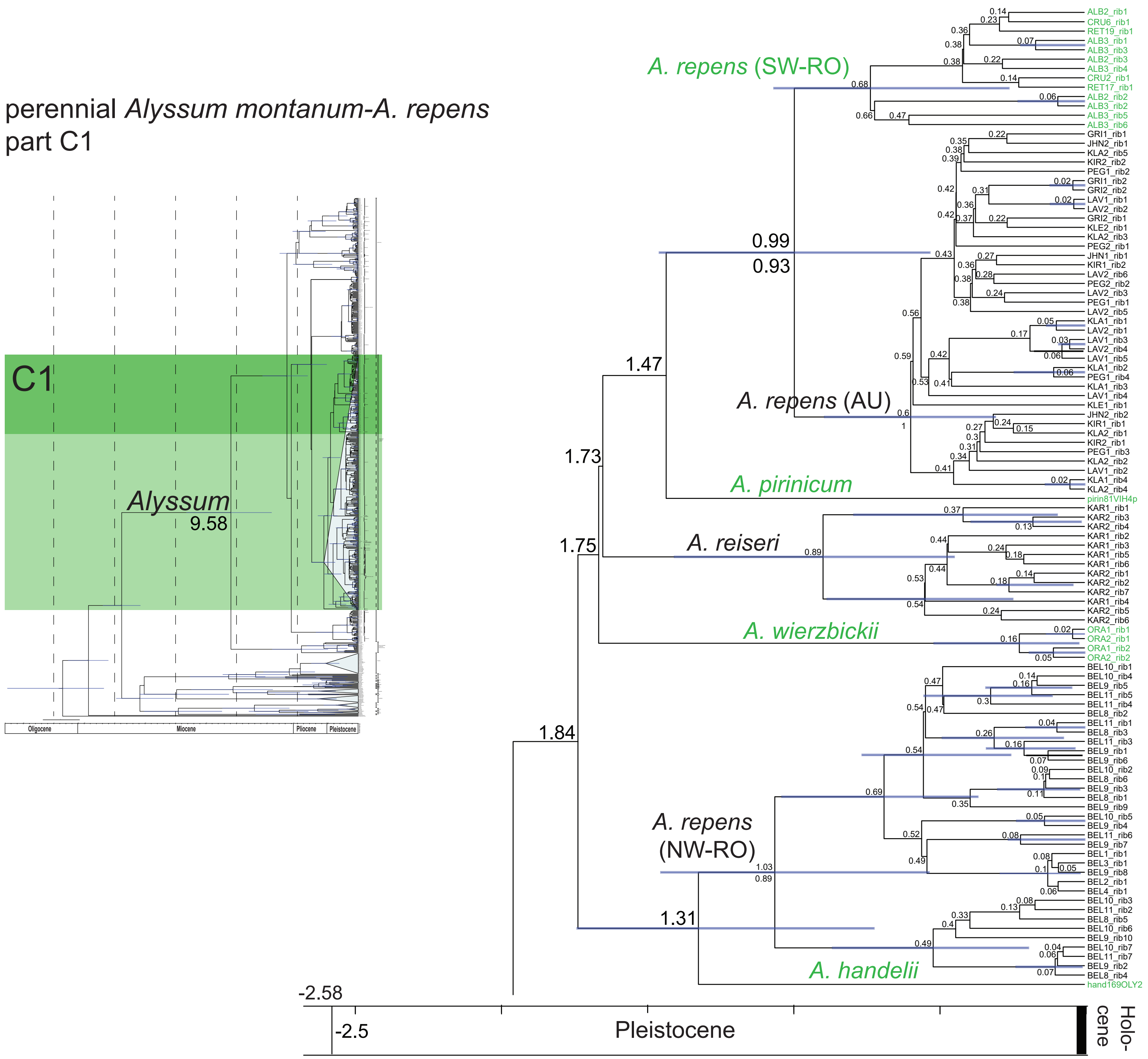

perennial *Alyssum montanum-A. repens*
part C1
C1
Alyssum
9.58
Oligocene
Miocene
Pliocene
Pleistocene
A. repens (SW-RO)
0.99
0.93
A. repens (AU)
1.47
1.73
A. pirinicum
A. reiseri
1.75
A. wierzbickii
1.84
A. repens
(NW-RO)
1.03
0.89
1.31
A. handelii
-2.58
-2.5
Pleistocene
Holo-
cene

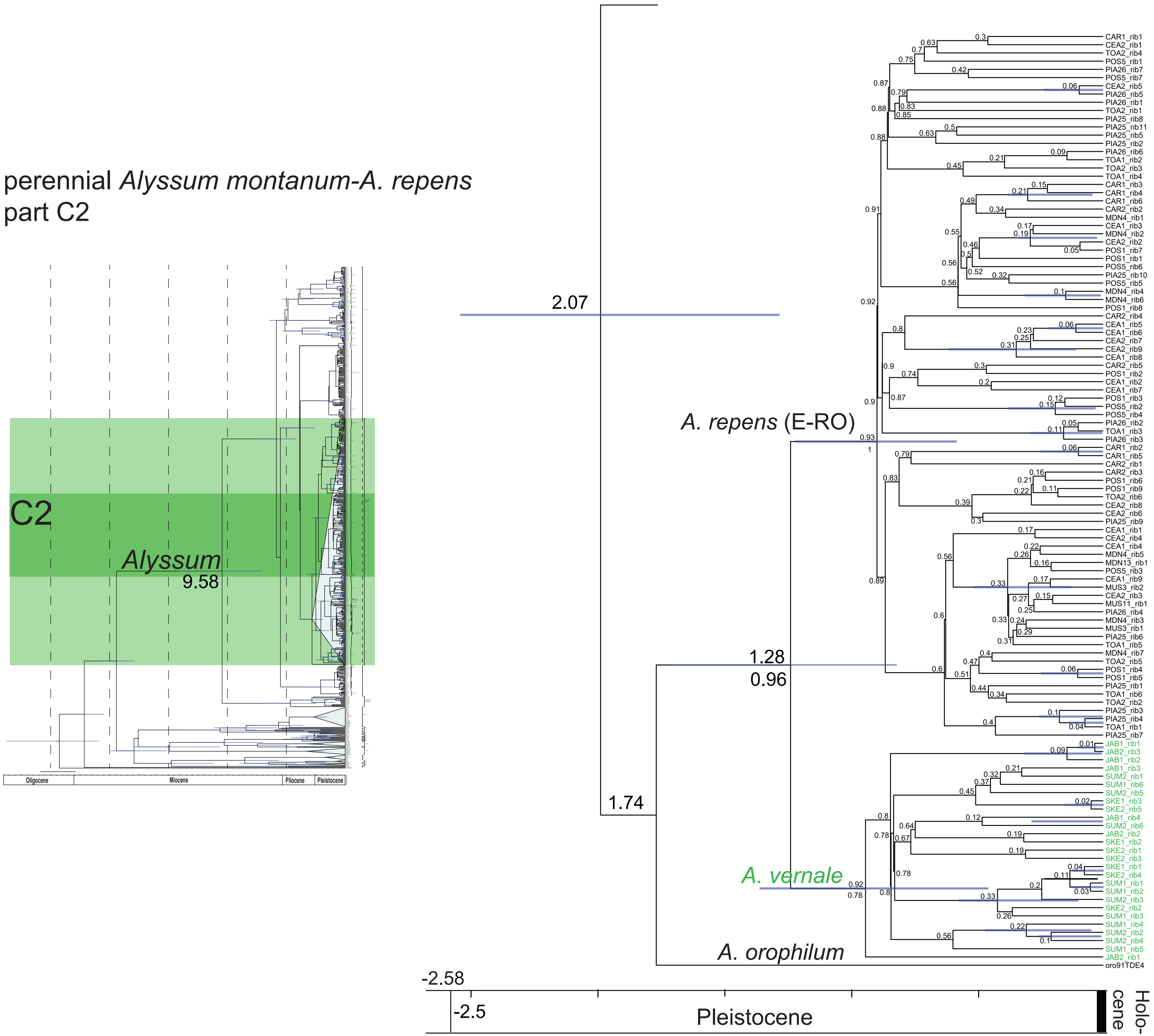
perennial Alyssum montanum-A. repens
part C2
C2
Alyssum
9.58
Oligocene
Miocene
Pliocene
Pleistocene
2.07
A. repens (E-RO)
1.28
0.96
1.74
A. vernale
A. orophilum
CAR1_rib1
CEA2_rib1
TOA2_rib4
POS5_rib1
PIA26_rib7
POS5_rib7
CEA2_rib5
PIA26_rib5
PIA26_rib1
TOA2_rib1
PIA25_rib8
PIA25_rib11
PIA25_rib5
PIA25_rib2
PIA26_rib6
TOA1_rib2
TOA2_rib3
TOA1_rib4
CAR1_rib3
CAR1_rib4
CAR1_rib6
CAR2_rib2
MDN4_rib1
CEA1_rib3
MDN4_rib2
CEA2_rib2
POS1_rib7
POS1_rib1
POS5_rib6
PIA25_rib10
POS5_rib5
MDN4_rib4
MDN4_rib6
POS1_rib8
CAR2_rib4
CEA1_rib5
CEA1_rib6
CEA2_rib7
CEA2_rib9
CEA1_rib8
CAR2_rib5
POS1_rib2
CEA1_rib2
CEA1_rib7
POS1_rib3
POS5_rib2
POS5_rib4
PIA26_rib2
TOA1_rib3
PIA26_rib3
CAR1_rib2
CAR1_rib5
CAR2_rib1
CAR2_rib3
POS1_rib6
POS1_rib9
TOA2_rib6
CEA2_rib8
CEA2_rib6
PIA25_rib9
CEA1_rib1
CEA2_rib4
CEA1_rib4
MDN4_rib5
MDN13_rib1
POS5_rib3
CEA1_rib9
MUS3_rib2
CEA2_rib3
MUS11_rib1
PIA26_rib4
MDN4_rib3
MUS3_rib1
PIA25_rib6
TOA1_rib5
MDN4_rib7
TOA2_rib5
POS1_rib4
POS1_rib5
PIA25_rib1
TOA1_rib6
TOA2_rib2
PIA25_rib3
PIA25_rib4
TOA1_rib1
PIA25_rib7
JAB1_rib1
JAB2_rib3
JAB1_rib2
JAB1_rib3
SUM2_rib1
SUM1_rib6
SUM2_rib5
SKE1_rib3
SKE2_rib5
JAB1_rib4
SUM2_rib6
JAB2_rib2
SKE1_rib2
SKE2_rib1
SKE2_rib3
SKE1_rib1
SKE2_rib4
SUM1_rib1
SUM1_rib2
SUM2_rib3
SKE2_rib2
SUM1_rib3
SUM1_rib4
SUM2_rib2
SUM2_rib4
SUM1_rib5
JAB2_rib1
oro91TDE4
-2.58
-2.5
Pleistocene
Holo-
cene

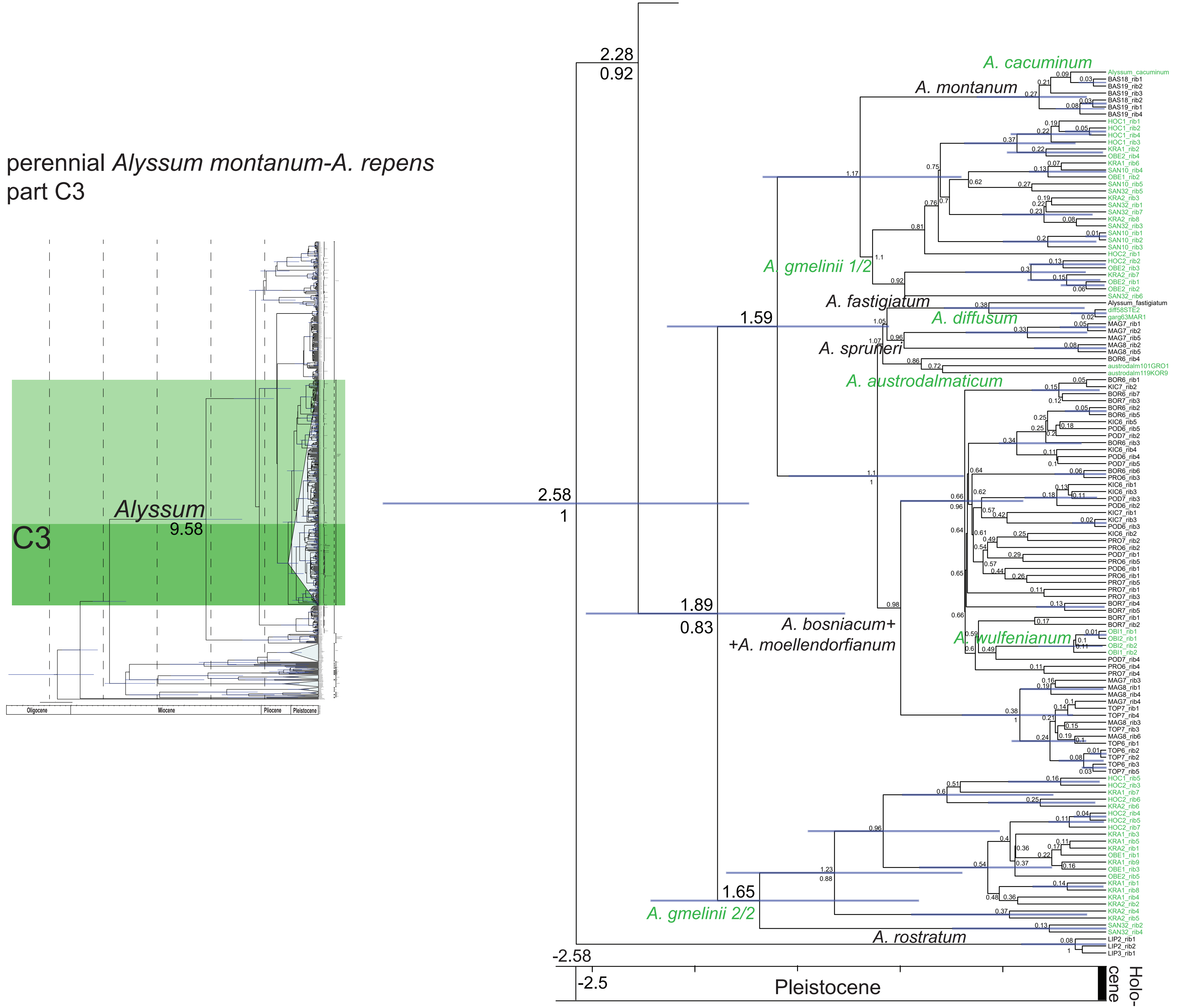

perennial *Alyssum montanum-A. repens*
part C3
C3
Alyssum
9.58
Oligocene
Miocene
Pliocene
Pleistocene
2.58
1
2.28
0.92
1.89
0.83
1.59
1.65
A. cacuminum
A. montanum
A. gmelinii 1/2
A. fastigiatum
A. diffusum
A. spruneri
A. austrodalmaticum
A. bosniacum+
+A. moellendorfianum
A. wulfenianum
A. gmelinii 2/2
A. rostratum
-2.58
-2.5
Pleistocene
Holo-
cene

Alyssum annuals + *Alyssum* sect. *Gamosepalum* and relatives
part D

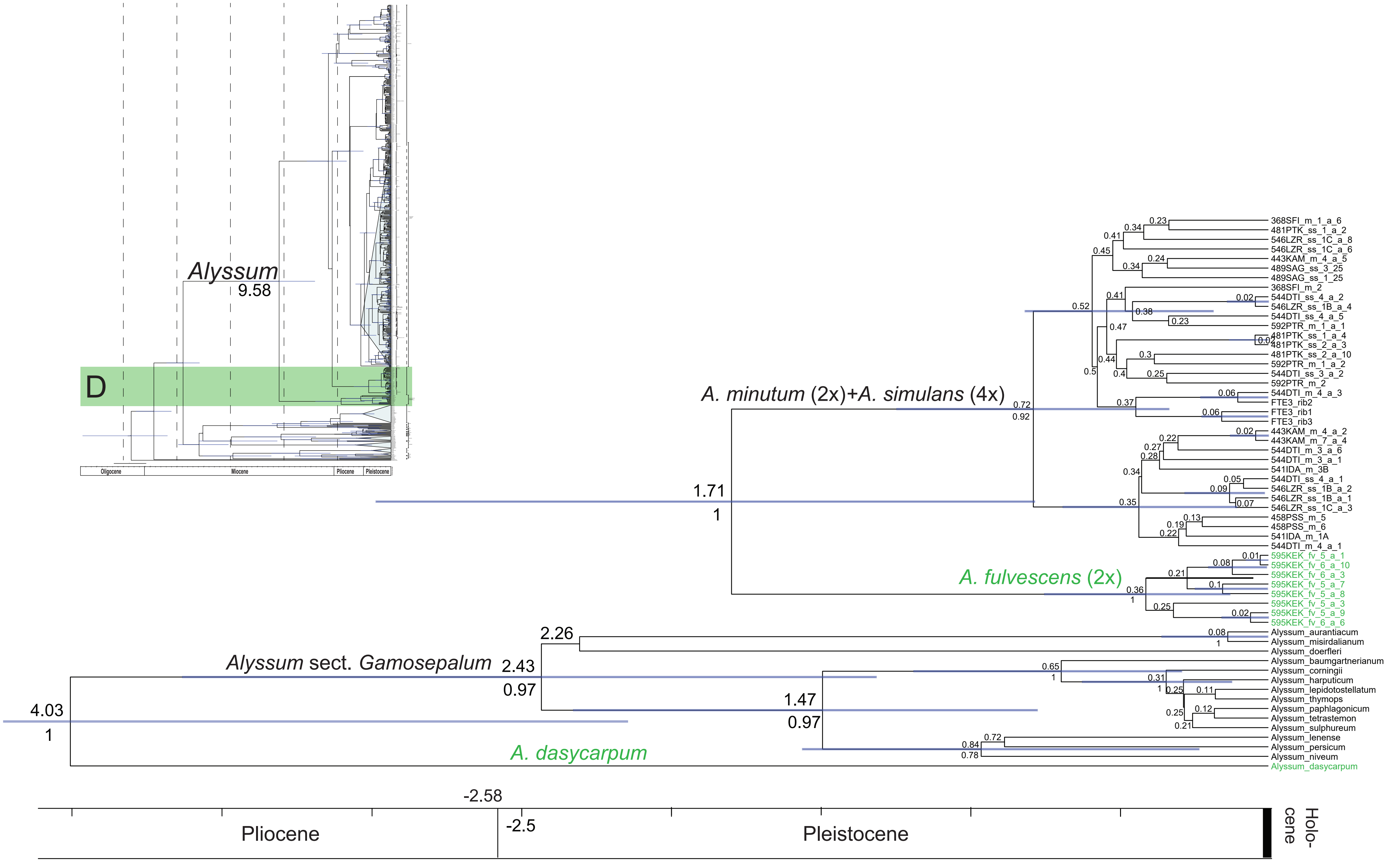

Supplement: Supplementary file 3 [file Data_Sheet_1.pdf]
